# Taxonomic status of the caturid genera (Halecomorphi, Caturidae) and their Late Jurassic species

**DOI:** 10.1098/rsos.221318

**Published:** 2023-01-11

**Authors:** Adriana López-Arbarello, Martin Ebert

**Affiliations:** ^1^ Department of Earth and Environmental Sciences, Paleontology and Geobiology, Ludwig-Maximilians-Universität München, Richard-Wagner-Str. 10, 80333 München, Germany; ^2^ GeoBio-Center, Ludwig-Maximilians-Universität München, Richard-Wagner-Str. 10, 80333 München, Germany

**Keywords:** Caturidae, *Caturus*, *Amblysemius*, *Strobilodus*, Jurassic

## Abstract

Caturids are among the best-known predatory ray-finned fishes of the Mesozoic. Although there is consensus about their sister-group relationship to Amiidae (Holostei, Amiiformes), their diversity, ingroup phylogenetic relationships and evolutionary history are still very poorly understood. Caturidae is currently restricted to *Caturus* and *Amblysemius*, each with two species. Among them, *C. furcatus* has become the wastebasket taxon for the group. Our revision of nearly 40 species based on the original descriptions, type and referred material led to significant results changing the picture of caturid diversity in the Late Jurassic. Four specific names are unavailable. Due to insufficient information in the original descriptions, lack of diagnostic features in the type material, or the complete lack of type material, 13 nominal species are nomina dubia. Two species currently considered junior synonyms represent distinct taxa. *Strobilodus giganteus* is removed from Caturidae. *Caturus cliftoni*, *Thlattodus* and *Ditaxiiodus* are tentatively referred to *Strobilodus*. The fossil record of Caturoidea is restricted to the Jurassic and earliest Cretaceous. The group apparently underwent significant diversification during the Late Jurassic, as indicated by the increase in the number of taxa and the dispersal of the group outside Europe, which had already begun in the Middle Jurassic.

## 1. Introduction

Caturids are among the most common and best-known predatory ray-finned fishes of the Late Jurassic. They have been mentioned frequently in the literature since their discovery in the early ninteenth century, including main reference books for vertebrate palaeontology and ichthyology (of those including fossils) [1–12]. They are currently accepted as the sister-group of Amiidae in the order Amiiformes (Neopterygii, Holostei, Halecomorphi) [13–18]. However, their diversity, ingroup phylogenetic relationships and evolutionary history are still very poorly understood.

The family Caturidae was named by Owen [19] to include *Caturus*, *Pachycormus*, *Saurostomus*, *Sauropsis* and *Furo* (=*Eugnathus*; the name *Eugnathus* Agassiz, 1839 [20] was preoccupied and replaced with *Furo* Gistel, 1848 [21]). Following Berg [22], *Pachycormus*, *Saurostomus* and *Sauropsis* are currently classified in a well-defined independent family Pachycormidae [23,24]. Nonetheless, after Owen [19] other taxa were added to the Caturidae and Lehman [1] listed a maximum of 11 genera in this family: *Allolepidotus*, *Callopterus*, *Caturus*, *Eoeugnathus*, *Eurycormus*, *Furo*, *Heterolepidotus*, *Lophiostomus*, *Macrepistius* and *Neorhombolepis*. *Osteorachis*, *Otomitla* and *Sinoeugnathus* have also been classified in Caturidae [25]. Patterson [25] removed from Caturidae the Triassic genera *Allolepidotus*, *Eoeugnathus* and *Sinoeugnathus*, which are currently placed in the Ionoscopiformes, and the Jurassic *Callopterus* and *Lophiostomus*, which are still considered halecomorphs of uncertain relationships. Patterson [25] also showed that *Eurycormus* was more closely related to teleosts than to *Caturus*, and Arratia *et al*. [26] later demonstrated that *Eurycormus* is a member of the Pholidophoriformes within Teleosteomorpha. Patterson notably improved the definition of the family Caturidae, but still thought that the remaining genera represented a paraphyletic group [25].

More recently, Lambers [14] presented a phylogenetic analysis, in which *Macrepistius*, *Heterolepidotus* and *Osteorachis* are more closely related to *Ophiopsis* (currently *Ophiopsiella* [27]) than to *Caturus*, and restricted the family Caturidae to *Caturus* and *Amblysemius* (a genus previously synonymized with *Caturus* that he revalidated 1 year earlier [28]). The more recent study of Grande & Bemis [13] supports the monophyly of Caturidae restricted to *Caturus* and *Amblysemius*, which is the sister-group of a monogeneric family Liodesmidae. These two families constitute the superfamily Caturoidea, which is the sister-group of the Amioidea, the lineage of the only living halecomorph *Amia calva* Linnaeus, 1766 [29].

The fossil record of Caturoidea is almost complete throughout the Jurassic (table 1), and the superfamily is particularly well represented in the Upper Jurassic deposits of England, France and Germany. Between 1833 and 1895, more than 30 Late Jurassic caturid species were named by different authors. Most of these taxa have been placed under synonymy or referred to other fish groups. Lambers [23,28] carried out the latest taxonomic revision of the caturids from the Upper Jurassic of the Solnhofen Archipelago, Germany, recognizing only four valid species arranged in two genera: *Caturus furcatus* and *C. giganteus*, and *Amblysemius pachyurus* and *A. bellicianus* [28]. However, around 20 nominal species of *Caturus* are placed in synonymy under one of these four taxa without a thorough revision of the original descriptions and type specimens. Hundreds of specimens distributed in many different collections in Europe and USA, which were previously identified in one or another of these numerous nominal species, are currently referred to the vaguely defined *Caturus furcatus*.

**Table 1.** Fossil record of Caturoidea (except *Liodesmus*). Only valid species are listed.

taxa	type specimen	stratigraphy	locality
Upper Triassic			
*Caturus insignis* (Kner, 1866)	TLM F.117	Norian	Seefeld, Austria
Lower Jurassic			
*Caturus heterurus* (Agassiz, 1839)	OUMNH P853343	Lower Lias	Lyme Regis, UK
*Caturus chirotes* (Agassiz, 1839)	NHMUK PV P.3642	Lower Lias	Lyme Regis, UK
*Caturus latipennis* (Agassiz, 1844)	NHMUK PV P.568	Lower Lias	Lyme Regis, UK
*Caturus agassizi* (Egerton, 1858)	NHMUK PV P.567	Lower Lias	Lyme Regis, UK
*Caturus smithwoodwardi* White, 1925	NHMUK PV P.11127	Toarcian	Holzmaden, Germany
Middle Jurassic			
*Caturus dartoni* Eastman, 1899	NMNH 4792	Bathonian	Hot Springs, South Dakota, USA
*Caturus porteri* Rayner, 1948	NHMUK PV P.29049	Callovian	Christian Malford, UK
Late Jurassic			
*Caturus deani* Gregory, 1923	AMNH FF 6371 (7930)	Oxfordian	Sheet La Palma, Cuba
*Strobilodus suchoides* (Owen, 1866)	NHMUK PV OR 41386	Kimmeridgian	Norfolk, UK
*Strobilodus impar* (Owen, 1866)	NHMUK PV OR 46318	Kimmeridgian	Oxfordshire, UK
*Strobilodus giganteus* Wagner, 1851	SNSB-BSPG 1953 I 579	Kimmeridgian	Kelheim and Painten, Germany
*Amblysemius granulatus* Münster, 1834	BSPG AS VII 1139	Kimmeridgian	Kelheim, Germany
*Caturus furcatus* (Agassiz, 1833)	NMP UC9/Uc83	Kimmeridgian	Kelheim, Germany
*Caturus macrurus* Agassiz, 1833	NMP Uc74	Kimmeridgian	Kelheim, Germany
*Amblysemius bellicianus* Thiollière, 1851	MHNL20015164	Kimmeridgian	Cerin, France
*Caturus* *latus* Münster, 1834	BSPG AS VII 263	Tithonian	Solnhofen, Germany
*Caturus ferox* Winkler, 1862	TM6901	Tithonian	Solnhofen, Germany
*Strobilodus cliftoni* (Woodward, 1895)	NHMUK PV P 6035	Tithonian	Isle of Portland, UK
*Catutoichthys olsacheri* Gouiric-Cavalli, 2016	MOZ-Pv 3645	Tithonian	Los Catutos, Neuquén, Argentina
Lower Cretaceous			
*Caturus purbeckensis* (Woodward, 1890)	NHMUK P PV-OR-46911	Berriasian	Swanage, Dorset, UK
*Caturus tenuidens* Woodward, 1895	NHMUK PV OR 40657 and NHMUK PV P 442a	Berriasian	Swanage, Dorset, UK
*Caturus* *tarraconensis* Sauvage, 1903*	MGB 514	Berriasian-lower Valanginian	El Montsec, Lérida, Spain

With the major aim to understand the evolutionary history of caturids, a few years ago we initiated the systematic revision of the Late Jurassic species of Caturidae. Although the detailed description and differential diagnoses of the valid taxa are still ongoing research, our thorough revision of the original descriptions and the type and referred material led to significant taxonomic results, which are summarized in the present contribution.

## 2. Material and methods

The taxonomic revision is based on the study of specimens and literature. The original literature was found either in the library of the SNSB-BSPG or in the online Biodiversity Heritage Library. Relevant text in German or French has been translated by the authors. Specimen photographs were taken with different digital cameras. In particular, detailed images were taken with a Jenoptik digital camera ProgRes C5 attached to a Leica Wild M80 stereomicroscope.

Published and unpublished artworks made for Louis Agassiz's ‘Recherches sur les Poissons Fossiles' are archived in the Geological Society Archive Catalogue, Geological Society, London, United Kingdom.

The International Code of Zoological Nomenclature (ICZN) is cited several times; it has been consulted online [30].

### 2.1. Abbreviations

Institutional Abbreviations. AMNH, American Museum of Natural History, New York, USA; CAM SM, Sedgwick Museum, Cambridge University, Cambridge, United Kingdom; JME, Jura-Museum Eichstätt, Germany; LDGSL, Deposited Series, Geological Society Archive Catalogue, Geological Society, London, United Kingdom; LMU, Ludwig Maximilians University, Munich, Germany; MB.f, Museum für Naturkunde, Berlin, Germany; MCZ, Museum of Comparative Zoology (Harvard University) Cambridge, Massachusetts, USA; MGB, Museo de Geología de Barcelona, Barcelona, Spain; MHNL, Muséum d'Histoire Naturelle de Lyon, France; MOZ, Museo Provincial Dr Prof. Juan Augusto Olsacher, Zapala, Neuquén, Argentina; NMNH, National Museum of Natural History (Smithsonian Institution), Washington, DC, USA; MPCA, Museo Provincial ‘Carlos Ameghino’, Cipolletti city, Río Negro province, Argentina; MRAC, Musée Royal de l'Afrique Centrale, Tervuren, Belgium; NHMUK, Natural History Museum, London, United Kingdom; NMP, National Museum Prague, Department of Palaeontology, Czech Republic; OUMNH, Oxford University Museum of Natural History, United Kingdom; SMNS, Staatliches Museum für Naturkunde Stuttgart, Germany; SNSB-BSPG, Staatliche Naturwissenschaftliche Sammlungen Bayerns–Bayerische Staatsammlung für Paläontologie und Geologie, Munich, Germany; TLM, Sammlungs- und Forschungszentrum der Tiroler Landesmuseen, Hall in Tirol, Austria; TM, Teylers Museum, Haarlem, The Netherlands.

## 3. Systematic palaeontology

Actinopterygii Cope, 1887 [31]

  Neopterygii Regan, 1923 [32]

  Holostei Müller, 1845 [33], *sensu* Huxley [34]

  Halecomorphi Cope, 1871 [35], *sensu* Grande [36]

  Amiiformes Hay, 1929 [37]

### 3.1. Caturoidea Owen, 1860 [19] *sensu* Grande & Bemis [13]

The superfamily Caturoidea is apomorphy-based defined by the following features: (1) the presence of sharply carinate acrodin tooth caps on the larger jaw teeth; (2) the presence of an extremely slender rod-like maxilla; (3) a relatively high number of branchiostegal rays (22 or more on each side); (4) shape of haemal spines broadly spatulate in the transverse plane; (5) the preural haemal and neural spines near the caudal peduncle region are strongly inclined to a nearly horizontal orientation [13].

#### 3.1.1. *Strobilodus* Wagner, 1851 [38]

**Figure 1.**
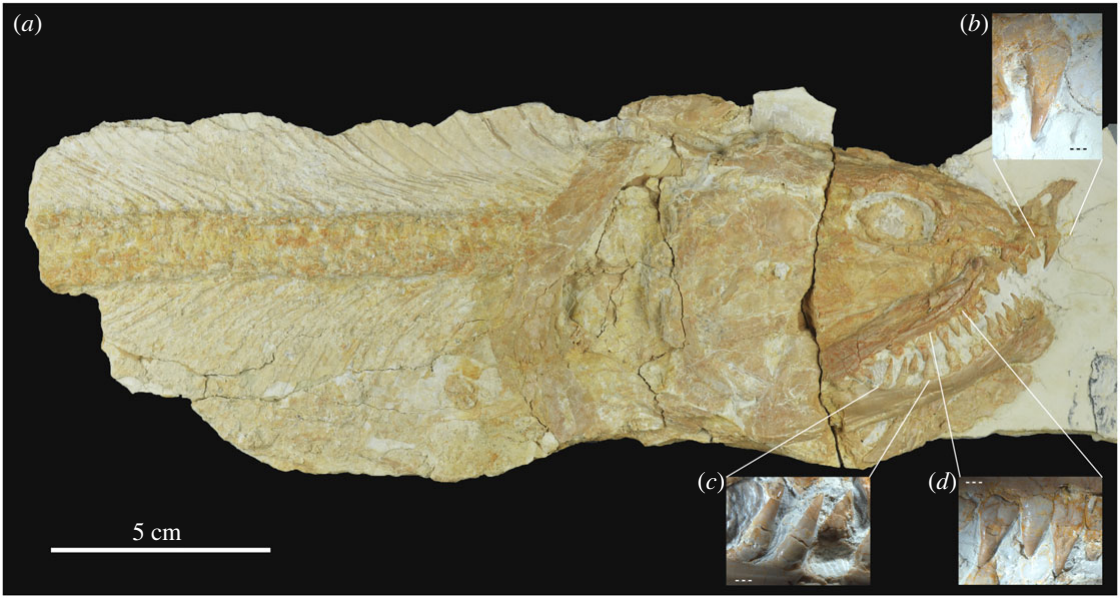
*Strobilodus giganteus* Wagner, 1851 [38]. Neotype SNSB-BSPG 1953 I 579 from Upper Jurassic (Kimmeridgian) lithographic limestones of Painten, Germany. (*a*) Photograph of the complete specimen by R. Winter (LMU); (*b*) detailed view of premaxillary fang; (*c*) detailed view of dentary fangs; (*d*) detailed view of maxillary fangs. (*b*–*d*) Photographs by A. López-Arbarello.

Type species—*Strobilodus giganteus* Wagner, 1851 [38], with neotype SNSB-BSPG 1953 I 579 (figure 1).

Discussion—The genus *Strobilodus* was erected by Wagner [38] for an imperfectly preserved but very distinct fish from the Upper Jurassic (upper Kimmeridgian [39]) lithographic limestones of Kelheim (most probably Kapfelberg), Germany, in the Munich palaeontological collection, which he named *Strobilodus giganteus*. Wagner's description of the species is rather extensive (electronic supplementary material, File), and he mainly distinguishes this taxon based on the very powerful dentition consisting of a series of strongly built cone-shaped teeth, which decrease in size towards the posterior end of the maxilla. He further described the lower jaw as narrow. Wagner accompanied his description with an illustration of the skull, pelvic bone and some disarticulated scales of the original specimen [38, pl. 2]. The type specimen is lost, but the description and illustration warrant the identification of the species which is well represented by specimen SNSB-BSPG 1953 I 579 from the locality of Painten close to Kelheim, Germany.

The very distinct shape of the maxilla (distinctly broader than in *Caturus* or *Amblysemius*), the relatively narrow and anteriorly tapering dentary, and the powerful conical teeth undoubtedly distinguish *Strobilodus* from *Caturus*. The marginal cutting teeth of *Caturus* have laterally compressed and sharply carinate acrodin caps. The marginal jaw bones of *Strobilodus* are garnished with strong fangs with conical caps. However, and despite recognizing the morphological differences, Woodward [40] placed the two genera under synonymy and the species has been treated as *Caturus giganteus* since then (e.g. [23,28]). Here, we not only revalidate *Strobilodus giganteus* as a distinct genus, but we also indicate other species that might also represent this very distinct taxon (§4) [32,33,35].

Wagner's [38] description and illustration of *Strobilodus giganteus*, as well as the proposed neotype, diverge from the apomorphy based diagnosis of Caturoidea on the morphology of the teeth and maxilla (features 1 and 2). The branchiostegal rays (character 3) are not preserved, but the haemal spines are transversally compressed, strongly inclined and tightly packed as indicated in characters 4 and 5. A thorough revision of the taxon and the study of its phylogenetic relationships are necessary to clarify its potential sister group relationship with other caturids. Pending such a study, *Strobilodus* is here removed from the Caturidae *sensu* Grande & Bemis [13] and it is tentatively placed in Caturoidea incertae sedis.

### 3.2. Caturidae Owen, 1860 [19] *sensu* Grande & Bemis [13]

#### 3.2.1. *Caturus* Agassiz, 1834 [40]

**Figure 2.**
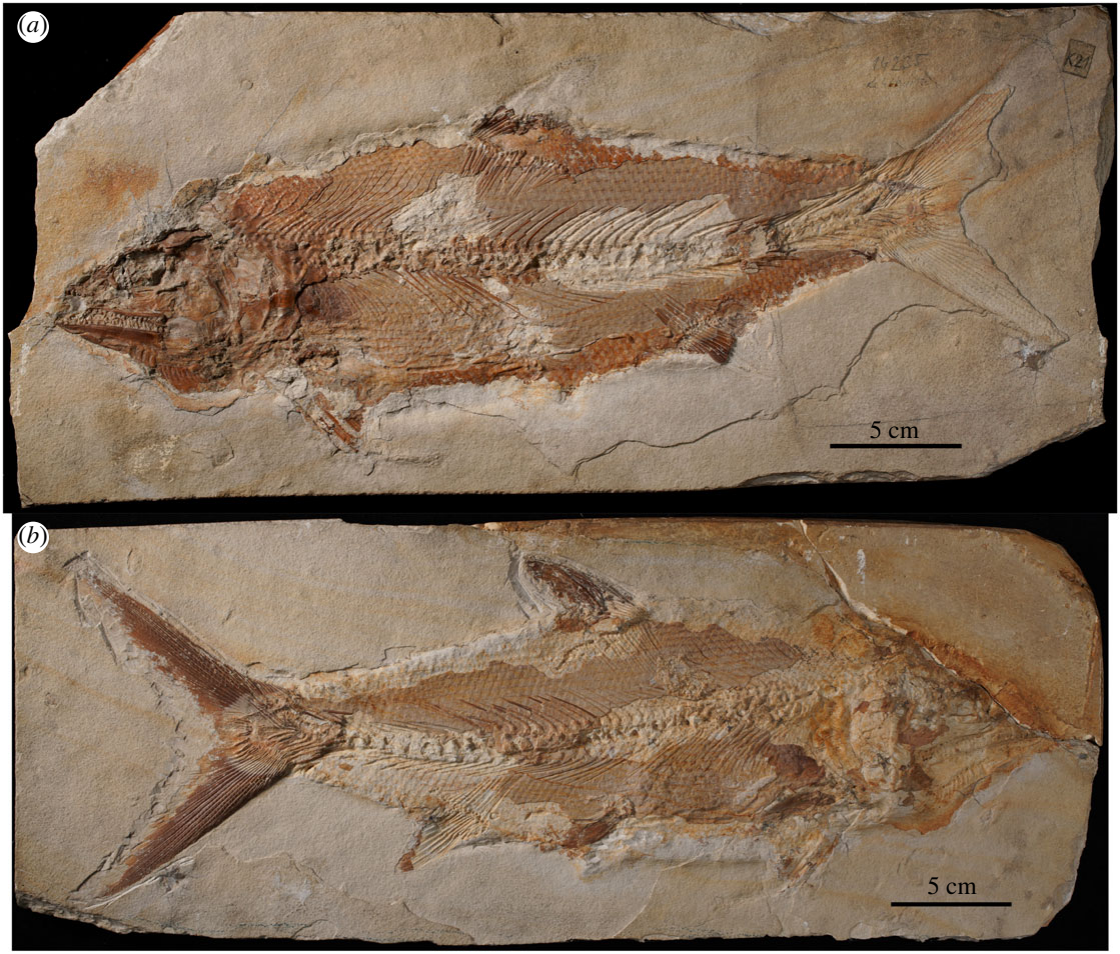
*Caturus furcatus* (Agassiz, 1833) [41]. Holotype specimen from Upper Jurassic (upper Kimmeridgian [39]) lithographic limestones of Kelheim, Germany. (*a*) Part NMP Uc9 and (*b*) counterpart Uc83. Photographs by A. López-Arbarello.

Type species—*Caturus furcatus* (Agassiz, 1833) [41] with holotype NMP Uc9 (counterpart Uc83) (figure 2).

Discussion—The name *Caturus* Agassiz, 1834 [42] was introduced as a nomen novum to replace *Uraeus* Agassiz, 1832 [43] because it was preoccupied by *Uraeus* Wagler, 1830 [44], which is a subgenus of the cobras *Naja* [45]. The latter name was erected by Agassiz, including two nominal species *Uraeus pachyurus* and *Uraeus gracilis*. None of these nominal species was accompanied with an illustration or sufficient description, and none of them was designated as the type species of the genus. Many years later, Woodward [40] presented *Caturus furcatus* (Agassiz, 1833) [41] as the type species of the genus, but this designation is incorrect because the type species of a genus must be chosen among the originally included taxa (ICZN Art. 67.2).

**Figure 3.**
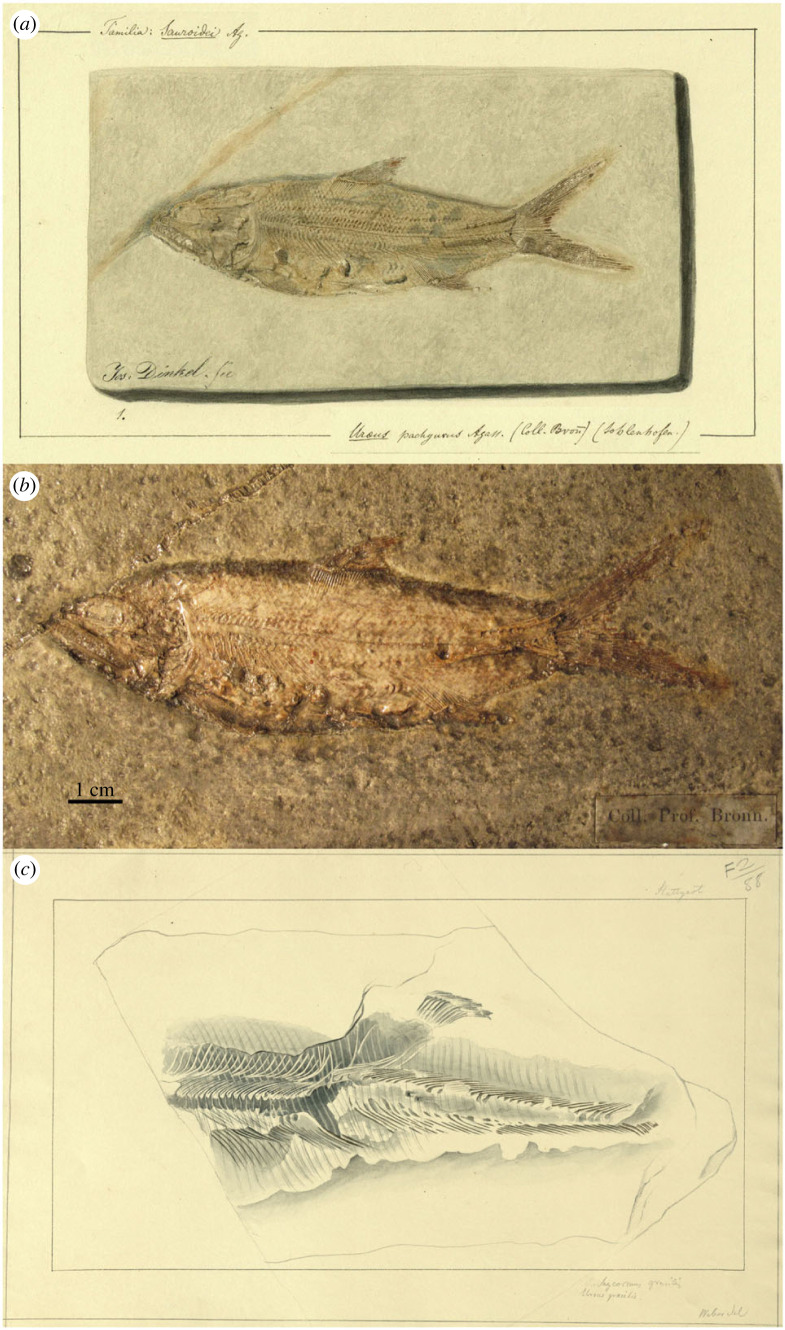
*Uraeus* Agassiz, 1832 [43]. (*a*) *Uraeus pachyurus* Agassiz, 1833 [41], LDGSL/614/2/79, illustration of the type specimen by Joseph Dinkel between 1832 and 1833. (*b*) *Uraeus pachyurus* Agassiz, 1833 [41], photograph of the holotype MCZ VPF-6262 from the Upper Jurassic (Tithonian) lithographic limestones of Solnhofen, Germany, by Ch. Byrd (MCZ). (*c*) Possible type specimen of *Uraeus gracilis* Agassiz, 1832 [43], represented in LDGSL/614/2/88, watercolour study by Charles Weber between 1831 and 1834.

Of the two species originally included in *Uraeus*, *U. pachyurus* is not an available name because it is not accompanied with a description, definition or indication (ICZN Art. 12.1). The simple references given by Agassiz for this species are not sufficient to constitute an indication (ICZN Arts. 12.2 and 12.3). Agassiz [43, p. 142] only wrote: ‘Von diesem Genus und zwar vom Solnhofer *Uraeus pachyurus* ist das schönste Exemplar, das ich je gesehen, in der Bronn'schen Sammlung in Heidelberg’ [Of this genus, and specifically of the Solnhofen *Uraeus pachyurus*, the most beautiful specimen I have ever seen is in the Bronn collection in Heidelberg]. Although the nominal species takes authorship and date *Uraeus pachyurus* Agassiz, 1833 [41] (figure 3*a,b*; see below), it is excluded from the taxa originally included in *Uraeus* Agassiz, 1832 [43].

Consequently, the type species of *Uraeus* Agassiz, 1832 [43] is *U. gracilis* by monotypy (ICZN Arts. 67.2). The morphological features indicated for *Uraeus gracilis* are not sufficient to distinguish a species: ‘*U. gracilis* AG. Sehr schlank, und bedeutend groß, ziemlich großschuppig’ [43, p. 142] [*U. gracilis* AG. Very slender, and significantly large, rather large-scaled]. In 1833, Agassiz [41] moved his *U. gracilis* to the new genus *Pachycormus*, including also *P. furcatus* and *P. macropterus*, adding the provenance of the species in the Lower Jurassic (Lias) of Baden-Württemberg, Germany. Although it has not been possible to locate the type specimen, there exists an unpublished illustration (LDGSL/614/2/88) possibly representing the fossil studied by Agassiz [41,43]. This artwork performed by C. Weber between 1831 and 1834 (v 3C) shows a fish labelled ‘*Pachycormus gracilis*, *Uraeus gracilis*', indicated ‘from Stuttgart’, but it is unclear if this is a reference to the locality or the collection. However, the reference probably refers to a natural history collection in Stuttgart, a predecessor of the Natural History Museum in Stuttgart (SMNS), because the Lower Jurassic outcrops of Baden-Württemberg do not reach the city of Stuttgart. The fossil represented in LDGSL/614/2/88 is very incomplete preserving only the middle body of a pachycormid-like fish without the skull and most fins, with only a portion of the dorsal fin preserved. The drawing was stored in a folder labelled ‘*Pachycormus* and *Sauropsis*' (both Pachycormidae). Based on the illustration or the brief original description, it is not possible to identify the fish at a lower taxonomic level, and it has been impossible to find the specimen in the SMNS collections (E. Maxwell 2022, personal communication). Therefore, although the brief and uninformative characterization of this species is acceptable as a definition in the light of ICZN Art. 12.1, the name *Uraeus gracilis* represents a nomen dubium.

The discovery of the overseen type species of *Uraeus* Agassiz, 1832 [43] now threatens the stability of the long-accepted names of *Caturus* and *Pachycormus* in their conventional meaning. In 1833, Agassiz [41] erected the name *Pachycormus*, including three species: *P. furcatus* Agassiz, 1833 [41], *P. macropterus* (Blainville, 1818) [46] and *P. gracilis* (= *Uraeus gracilis* Agassiz, 1832 [43]). Therefore, *Pachycormus* is an objective junior synonym of *Uraeus*. In 1834, Agassiz [42] replaced his *Uraeus* with the name *Caturus* and, thus, the latter is the objective senior synonym of *Pachycormus*. All these generic names have the same type species *Uraeus gracilis* Agassiz, 1832 [43], which is a nomen dubium. To preserve the stability of the well-established names *Pachycormus*, with type species *P. macropterus*, and *Caturus*, with type species *C. furcatus*, an application to the International Commission on Zoological Nomenclature will be submitted to the Bulletin of Zoological Nomenclature. Pending the resolution of this case, we keep the broadly accepted designation of *Caturus furcatus* as the genotype [40].

#### 3.2.2. *Amblysemius* Agassiz, 1844 [46]

**Figure 4.**
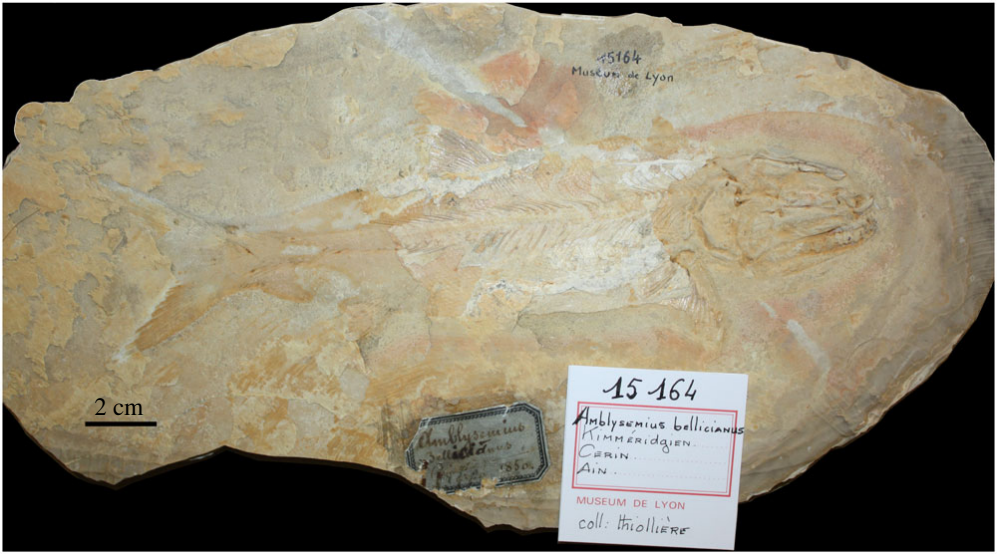
*Amblysemius bellicianus* Thiollière, 1851 [47]. Holotype MHNL20015164, collection Thiollière, from the Upper Jurassic (upper Kimmeridgian­ [48]) lithographic limestones of Cerin, France. Photograph by M. Ebert.

Type species—*Amblysemius bellicianus* Thiollière, 1851 [47], with holotype MHNL20015164 (figure 4).

Discussion—The generic name *Amblysemius* was erected by Agassiz in 1844, recognizing its similitude with *Caturus* but providing several features distinguishing the new nominal taxon from the latter. These features constitute a description or definition in the sense of ICZN Art. 12, thus making the name *Amblysemius* Agassiz, 1844 [49] available. However, the content of the genus is unknown. In the nomenclatural act of erecting the generic name *Amblysemius*, Agassiz [49, vol. 2, part 2, p. 119] did not name or indicate a species representing the content of the genus. Later, though with the same publication date, Agassiz [49, vol. 2, part 2, p. 165] listed ‘*Amblysemius gracilis*.— Oolite de Northampton’. Without description, definition or indication and, thus, failing to conform to ICZN Art. 12, *Amblysemius gracilis* Agassiz, 1844 [49] is a nomen nudum (unavailable name).

Resembling the case of the holotype of *Uraeus gracilis*, there exists an unpublished artwork of a specimen labelled *Amblysemius gracilis* (LDGSL/614/2/103a). The illustration was performed by Charles Weber for Louis Agassiz's ‘Recherches sur les Poissons Fossiles' between 1833 and 1843/1844, but it is not accompanied by any information on the provenance of the fossil and there is no evidence that it could be the fish from the Oolite of Northampton examined by Agassiz. Although *Amblysemius* stayed bound to it, the nominal taxon *Amblysemius gracilis* was never described or figured remaining obscure [50,51]. Woodward [40, p. 350] wrote: ‘Nothing is known of the so-called *Amblysemius gracilis* (…) from the Oolite of Northampton, said to be closely related to *Caturus* (…)’.

In 1851 Thiollière [47] erected the specific name *Amblysemius bellicianus* for a fish from the Upper Jurassic (upper Kimmeridgian [48]) lithographic limestones of Cerin, France (original spelling by Thiollière in the same publication corrected ‘*bellovacinus*’ on page 38 to ‘*bellicianus*’ in a footnote on page 58 and in a section ‘ERRATA’ on page 76). The name *A. bellicianus* Thiollière, 1851 [47] is accompanied with a description and, thus, it is available (ICZN Art. 12). Although there is no indication or illustration of a type specimen, the specimen MHNL20015164 (figure 4) belonging to the collection Thiollière in the Museum de Lyon and labelled ‘*Amblysemius bellicianus*', with the specific epithet corrected and the year 1850 on the label, is most probably the fossil on which Thiollière based this species. Therefore, in agreement with ICZN Arts. 72.2 and 72.4.1 specimen MHNL20015164 is here fixed as the holotype of *Amblysemius bellicianus* Thiollière, 1851 [47]. MHNL20015159 is the counterpart of MHNL20015164. It is mainly a cranium which was prepared with acid.

Since the species first included in *Amblysemius*, *A. gracilis* Agassiz, 1844 [49], is a nomen nudum, *A*. *bellicianus* Thiollière, 1851 [47] is the first available name expressly included in *Amblysemius* Agassiz, 1844 [49], and, according to ICZN Art. 67.2.2, it must be accepted as the type species of the genus.

It is worth noting that there has been tremendous confusion about the definition of *Amblysemius* Agassiz, 1844. Saint-Seine [52] questioned the validity of *A. gracilis* Agassiz, 1844 [49] and considered ‘*A*.’ *bellicianus* Thiollière, 1851 [47] as a species of *Caturus*. However, Saint-Seine's [52] description of *Caturus bellicianus* is mainly based on the specimen MHNL20015172, which he erroneously took for the holotype. Thiollière described and illustrated MHNL20015172 under the name ‘*Caturus elongatus* Agassiz’ [47, pp. 34–35; 53, p. 18, pl. VII, fig. 2].

Lambers [23,28] is very confusing about type species and type specimens. To begin with, he indicates ‘*Amblysemius gracilis* (Agassiz 1833)’ as the type species of the genus *Amblysemius* [23, p. 136] instead of *Amblysemius gracilis* Agassiz, 1844 [49], apparently mixing up the name with *Uraeus gracilis* Agassiz, 1832 [43], which is a different nominal taxon (see above). Lambers later notes ‘*A. pachyurus* (Agassiz, 1832)’ from the Solnhofen Archipelago, Germany, as the type species of *Amblysemius* in the systematic palaeontology section, but otherwise indicates *A. gracilis* as the type of the genus elsewhere in the text [28, pp. 91, 97]. Although he recognizes ‘*A. pachyurus* (Agassiz, 1832)’ and ‘*A. bellicianus* Thiollière, 1851’ as valid taxa, he did not examine the corresponding type material. He acknowledges not knowing the type specimen of *Caturus pachyurus*, which he considered lost, and his taxonomic conclusions on ‘*A. pachyurus*’ are based on the holotype of *Caturus granulatus* Münster, 1842 (SNSB-BSPG AS VII 1139) from Kelheim, Bavaria, Germany, arguing its overall similarity with specimens labelled *C. pachyurus* (SNSB-BSPG AS I 1249 from Eichstätt and NHMUK PV P 44900 from Solnhofen, both Bavaria, Germany). On the other hand, Lambers [23,28] based his diagnosis and description of his ‘*A. bellicianus*’ on the specimen that Thiollière referred to *Caturus elongatus*, MHNL20015172 from Cerin, France, which was erroneously taken as the holotype of ‘*A.*’ *bellicianus* by Saint-Seine [52], and the then still unstudied specimen SNSB-BSPG 1969 XVII 50 from Zandt, Bavaria, Germany.

## 4. Late Jurassic nominal species of Caturoidea

In this section, we will discuss the taxonomic status of each of the Late Jurassic nominal species of Caturoidea, based on our study of the literature, type series and collections research. The nominal species are ordered chronologically.

### 4.1. *Uraeus pachyurus* Agassiz, 1832 [42]

Nomen nudum (see §3.2.1).

### 4.2. *Uraeus nuchalis* Agassiz, 1833 [41]

The type specimen is unknown, and the original and only known description is insufficient to validate the taxon: ‘*U. nuchalis*. Nacken wulstig mit grösseren Schuppen; Körper gegen den Schwanz schmäler werdend. Solenhofen’ [41, 477] [*U. nuchalis*. Nape bulging with larger scales; body narrowing towards the tail. Solnhofen].

The nominal taxon has been considered a junior synonym of *Caturus furcatus* [40,49,54], but it is not possible to either confirm or refute such hypothesis and the taxon is here regarded as a nomen dubium.

### 4.3. *Uraeus pachyurus* Agassiz, 1833 [41]

The original description is not sufficient to validate the taxon: ‘*U. pachyusus*. Schwanz dick; Körper in gleicher Flucht; Solenhofen’ [41, 477] (specific epithet misspelled) [*U. pachyurus*. Tail thick; body in equal alignment; Solnhofen]. An unpublished illustration labelled *Uraeus pachyurus* of a complete specimen from Solnhofen, Germany, which was part of the Bronn Collection in Heidelberg, has been found in the Geological Society Archive Catalogue LDGSL/614/2/79 (figure 3*a*). Although it was finally not included, the illustration by Joseph Dinkel was made for Agassiz's ‘Recherches sur les Poissons Fossiles'. Furthermore, the specimen has been located in the Museum of Comparative Zoology with collection number MCZ VPF-6262 (figure 3*b*), and additional information accompanying the specimen supports the hypothesis that this is most probably the fossil meant to represent *U. pachyurus* by Agassiz [43]. Therefore, according to the Arts. 72.4.1.1 and 73.1.2 of the Code, specimen MCZ VPF-6262 is the holotype of *Uraeus pachyurus* Agassiz, 1833.

The nominal taxon *Caturus* (*Uraeus*) *pachyurus* has been considered as a distinct species of *Caturus* by Wagner [54], Woodward [40] and Heineke [55], or as the type species of *Amblysemius* by Lambers [28]. However, none of those authors examined the type specimen, which is complete but rather poorly preserved. Because of the deficiency of the original description and the absence of diagnostic features in the holotype MCZ VPF-6262, this nominal taxon is considered a nomen dubium.

### 4.4. *Uraeus macrocephalus* Agassiz, 1833 [41]

Holotype SNSB-BSPG AS I 1134 from Solnhofen, Germany. The species was first referred to the genus *Pholidophorus* and split in two taxa, *Ph. macrocephalus* and *Ph. uraeoides* [41], and later distinguished as a new taxon *Siemensichthys macrocephalus* [56], including *Ph. uraeoides* as a junior synonym.

### 4.5. *Uraeus microlepidotus* Agassiz, 1833 [41]

Holotype SNSB-BSPG AS V 11a,b, from Eichstätt (holotype according to Lambers [57]). The species was referred to *Eugnathus* by Agassiz [58] and is currently accepted as ‘*Furo*’ *microlepidotes* [57,59,60].

### 4.6. *Uraeus macrurus* Agassiz, 1833 [41]

**Figure 5.**
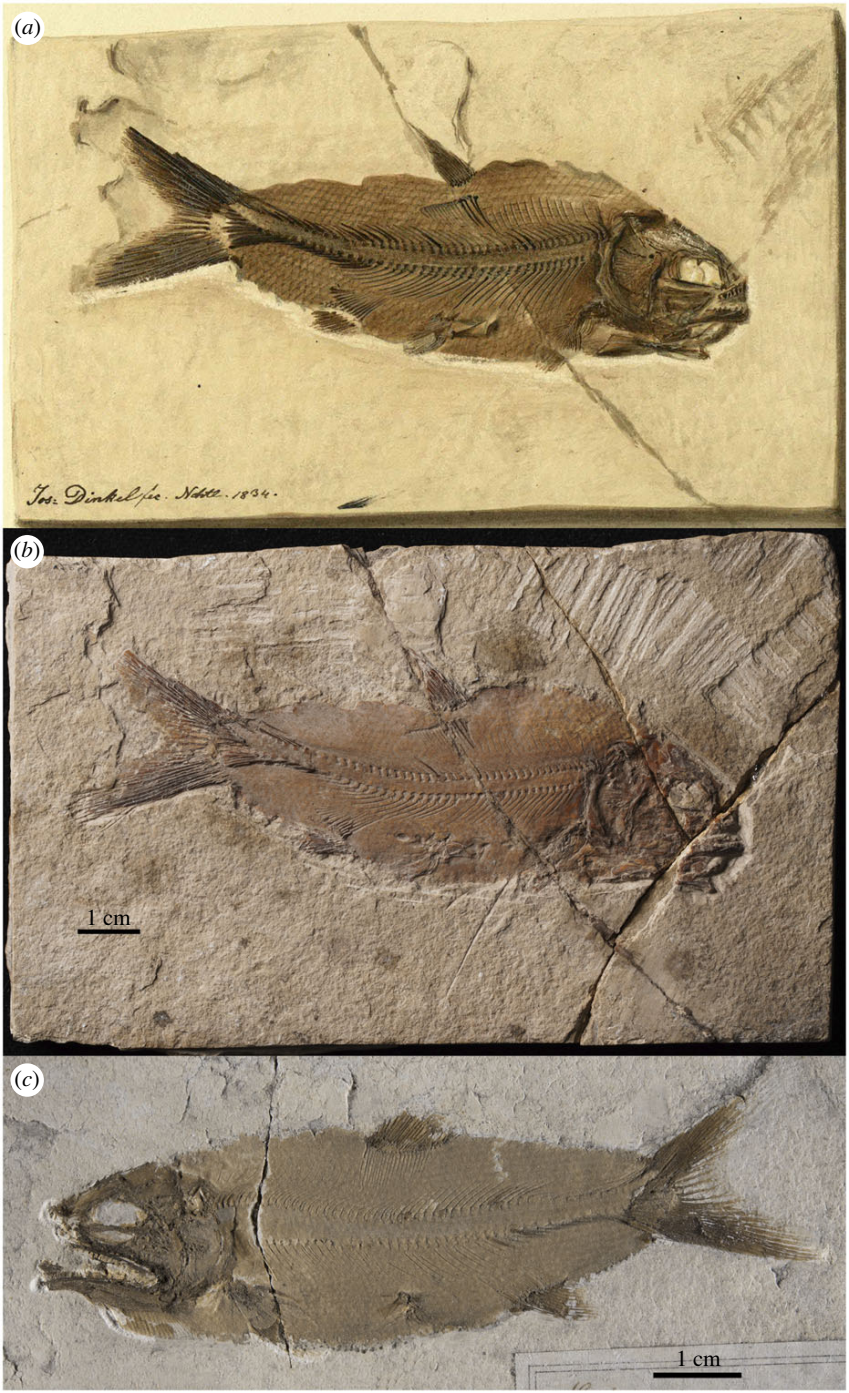
*Uraeus macrurus* Agassiz, 1833 [41]. (*a*) LDGSL /614/2/71, illustration of the type specimen by Joseph Dinkel in 1834. (*b*) Lectotype NMP Uc74 from the Upper Jurassic (upper Kimmeridgian [39]) lithographic limestones of Kelheim, Germany, photograph by B. Ekrt (NMP). (*c*) MB.f.12850, holotype of *Caturus obovatus* Münster, 1842 [61], photograph by H. J. Götz (MB).

The original and only available description is very unsatisfactory: ‘*U. macrurus*. Klein mit verhält. sehr grossem tiefgabeligem Schwanz’ [41, 477] [*U. macrurus*. Small with a proportionally very large deeply forked tail]. Three unpublished illustrations of specimens labelled ‘*Uraeus macrurus*’, two by Charles Weber in 1831 (LDGSL/614/2/72 and LDGSL/614/2/73) and one by Joseph Dinkel in 1834 (LDGSL /614/2/71; figure 5*a*), might represent the type specimens. The first two of these specimens from Solnhofen were in the Paleontological Museum in Munich and were probably destroyed during WWII. The third specimen from Kelheim (most probably Kapfelberg), Germany has been located in Prague with collection number NMP Uc74 (figure 5*b*). Therefore, according to the Arts. 72.4.1.1 and 74.7 of the Code, specimen NMP Uc74 is hereby designated lectotype of *Uraeus macrurus* Agassiz, 1833.

The species *Caturus* (*Uraeus*) *macrurus* has been considered as a junior synonym of *Caturus furcatus* [23,28,40]. The lectotype is significantly smaller than the holotype of *Caturus furcatus* and shows some morphological differences that might be due to ontogenetic variation. However, by examining the type material we have not yet been able to confirm either the synonymy or the validity of the taxon. Detailed study of this material will clarify the potential validity of *C. macrurus* Agassiz, 1833.

### 4.7. *Caturus furcatus* (Agassiz, 1833) [41]

Original name *Pachycormus furcatus*. Holotype NMP Uc9 (counterpart Uc83) (figure 2), from the Upper Jurassic (upper Kimmeridgian [39]) lithographic limestones of Kelheim (most probably Kapfelberg), Germany. The species was transferred to *Caturus* by Agassiz [62]. As explained above (§3.2.1), for the time being it remains the type species of the genus.

### 4.8. *Caturus elongatus* Agassiz, 1834 [62]

**Figure 6.**
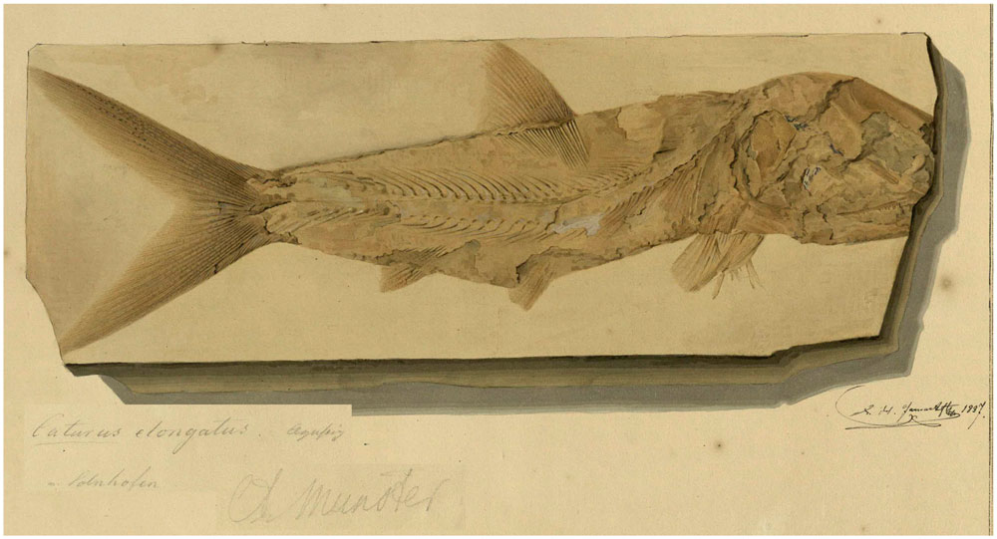
*Caturus elongatus* Agassiz, 1834 [62], LDGSL/614/2/67, illustration of the type specimen by S. H. Jarwart in 1837.

The nominal species is accompanied with a brief description: ‘*Caturus elongatus* Agass. (*Uræus elongatus* dans les collections que j'ai étiquetées.) Corps plus élancé que dans les autres espèces du genre. C. très-grande, très-fourchue. A. très -petite, étroite et courte. D. large, ayant un grand nombre de petits rayons dans son bord antérieur. Solenhofen’ [62, p. 13] [*Caturus elongatus* Agass. (*Uræus elongatus* in the collections I have labelled.) Body more slender than in the other species of the genus. Caudal fin very large, very forked. Anal fin very small, narrow and short. Dorsal fin broad, having a large number of small rays in its anterior margin. Solenhofen]. A watercolour study by Sixtus Heinrich Jarwart in 1837 (LDGSL/614/2/67) of a complete specimen from Solnhofen, Germany, in the collection of Georg Graf von Münster which became part of the collection of the SNSB-BSPG, shows a very imperfectly preserved fish that matches the specimen described as the original exemplar by Wagner [54] (figure 6). Unfortunately, it has not been possible to locate the fossil which was probably destroyed during WWII.

*Caturus elongatus* has been regarded as a junior synonym of *Caturus pachyurus* [23,40], *Amblysemius pachyurus* [23,28], or *Amblysemius bellicianus* [23,28]. In the absence of a type specimen and considering the deficient original description, neither the potential synonymy nor the validity of the taxon can be confirmed and the species is here regarded as a nomen dubium.

### 4.9. *Caturus maximus* Agassiz, 1834 [62]

The original description is not sufficient to distinguish the taxon: ‘*Caturus maximus* Agass. (*Uræus maximus* Collect.) Remarquable par le prolongement considérable des rayons extérieurs des deux lobes de la C., qui fait ressembler cette nageoire à celle des Coryphènes. Solenhofen’ [62, p. 13] [*Caturus maximus* Agass (*Uræus maximus* Collect.) Remarkable by the considerable extension of the external rays of the two lobes of the caudal fin, which makes this fin resemble that of the Coryphaenes. Solenhofen]. There are two unpublished artworks representing three specimens (LDGSL/614/2/74 and LDGSL/616/2/21/1), which are labelled with this nominal species. The three specimens are from Solnhofen and were part of the collection of Georg Graf von Münster.

**Figure 7.**
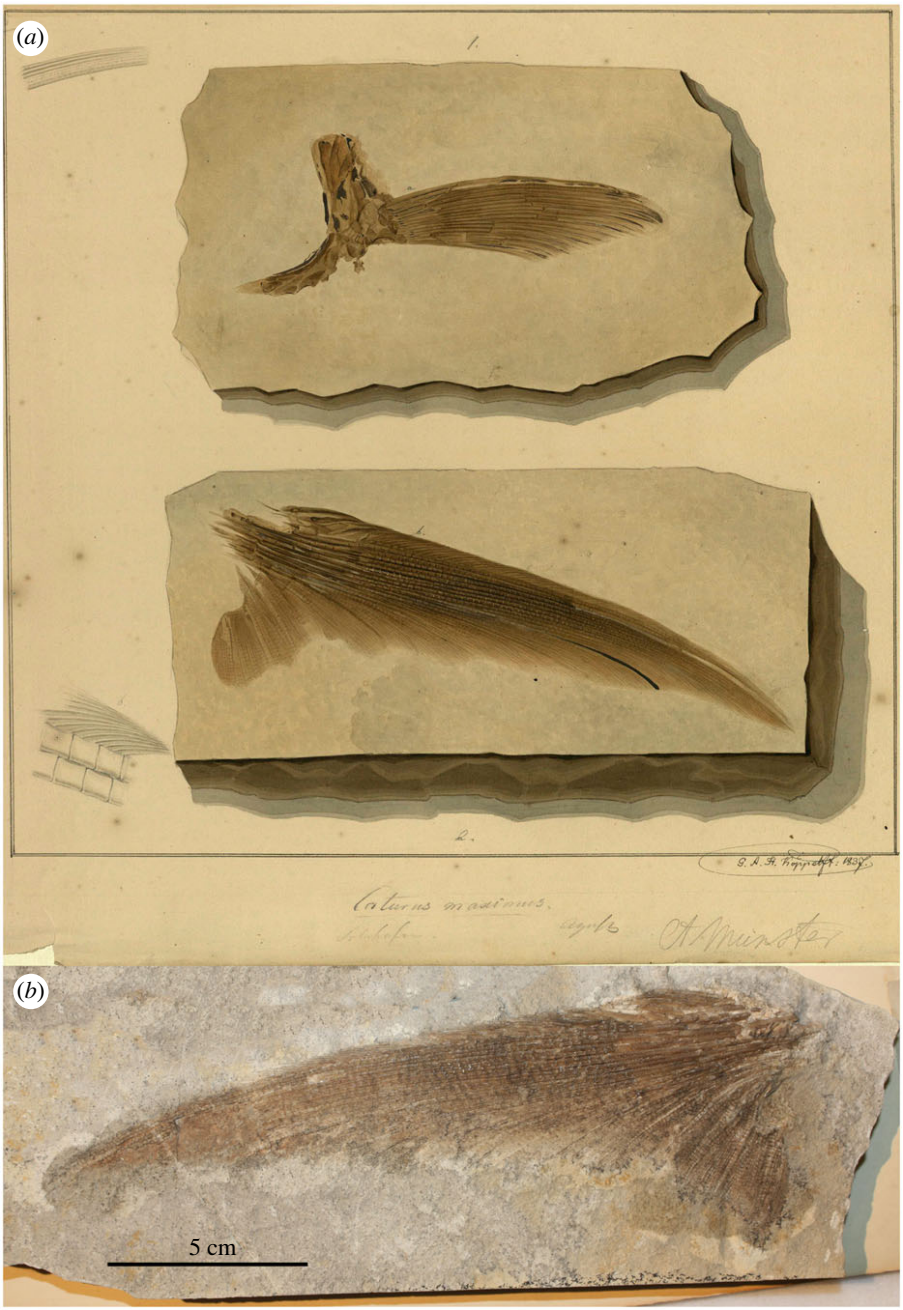
*Caturus maximus* Agassiz, 1834 [62]. (*a*) LDGSL/614/2/74, illustration by G. A. H. Köppel in 1837 of two specimens referred to *C. maximus*. (*b*) CAM SM 11115 from the Upper Jurassic (Tithonian) lithographic limestones of Solnhofen, Germany, corresponding to the counter slab of the partial caudal fin represented in the lower drawing of (*a*), photograph by M. Ebert.

The artwork LDGSL/614/2/74 (figure 7*a*), drawn by G. A. H. Köppel in 1837, includes a partial pectoral girdle and fin (upper drawing), and a partial caudal fin. Specimen CAM SM 11115 (figure 7*b*) is the counter slab of the fragment of caudal fin in the lower illustration. However, Agassiz [62] describes the two lobes of the caudal fin and, therefore, CAM SM 11115 cannot be identified with the type specimen of *C. maximus*.

The illustration LDGSL/616/2/21/1 represents the specimen NHMUK PV P 3583, which is a well preserved postcranium, including the almost complete caudal fin. This specimen fits perfectly the description of *Pholidophorus maximus* Agassiz, 1844 [63], which is a different taxon. *Pholidophorus maximus* has been referred to *Heterolepidotus* by Woodward [40], but direct examination of the specimen confirms that NHMUK PV P 3583 represents a ‘pholidophorid’ and, based on the characteristically finely serrated ganoid scales, the numerous small fringing fulcra and the large distinct dorsal precaudal scute, it can be referred to the Ankylophoridae Gaudant, 1978 [64] (M.E. 2022, personal observation).

Therefore, NHMUK PV P 3583 is undoubtedly the holotype of *Pholidophorus maximus* Agassiz, 1844 [63], and the holotype of *Caturus maximus* Agassiz, 1834 [62] is unknown. Although *C. maximus* has been considered as a junior synonym of *C. furcatus* [23,28,40], the very incomplete original description does not provide evidence to either confirm or reject this synonymy and the species is regarded as a nomen dubium.

### 4.10. *Caturus microchirus* Agassiz, 1834 [62]

**Figure 8.**
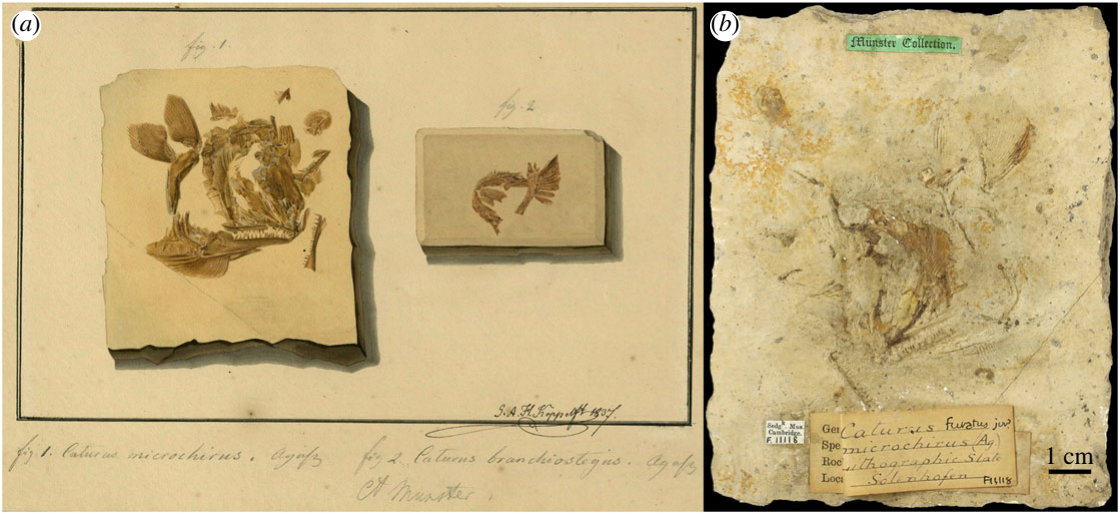
*Caturus microchirus* Agassiz, 1834 [62] and *C. branchiostegus* Agassiz, 1834 [59]. (*a*) LDGSL/614/2/75, illustration of the type specimen of *C. microchiurus* (‘fig. 1’ to the left) and holotype specimen of *C. branchiostegus* (‘fig. 2’ to the right) by G. A. H. Köppel in 1837; (*b*) holotype of *C. microchiurus*, CAM SM 11118, from the Upper Jurassic (Tithonian) lithographic limestones of Solnhofen, Germany, representing the counter slab of the original type specimen figured in (*a*), photograph by M. Friedman (University of Michigan).

The original description is insufficient: ‘*Caturus microchirus* Agass. (*Uræus microchirus* Collect.) P. à base large, mais a rayons courts et minces, le premier excepté; rayons branchiostègues antérieurs courts et étroits, insensiblement plus longs et plus larges, au nombre de vingt-quatre à vingt-cinq. Dents de la mâchoire inférieure plus éloignées et plus grandes que celles de la mâchoire supérieure. Solenhofen’ [62, pp. 13–14] [*Caturus microchirus* Agass. (*Uræus microchirus* Collect.) Pectoral fins with a broad base, but with short, slender rays, except the first; anterior branchiostegal rays short and narrow, gradually longer and wider, twenty-four to twenty-five in number. Teeth of the lower jaw further apart and larger than those of the upper. Solenhofen]. Specimen CAM SM 11118 is the counterpart of the specimen from the collection of Georg Graf von Münster which is illustrated in LDGSL/614/2/75 (figure 8), watercolour artwork by G. A. H. Köppel in 1837. Since the illustrated specimen is lost, its counterpart CAM SM 11118 is here fixed as the holotype of *Caturus microchirus* Agassiz, 1834.

Though *Caturus microchirus* has been considered as a junior synonym of *C. furcatus* [23,28,40], the holotype is too incomplete and shows no distinct feature allowing neither to confirm this synonymy nor to validate the nominal species, which must be treated as a nomen dubium.

### 4.11. *Caturus branchiostegus* Agassiz, 1834 [62]

The original description is insufficient to distinguish the taxon: ‘*Caturus branchiostegus* Agass. (*Uræus branchiostegus* Collect.) Je n'en connais qu'une mâchoire inférieure avec les rayons branchiostègues. Les dents sont rapprochées, la mâchoire courte; les rayons branchiostègues antérieurs plus larges que les suivans. Solenhofen’ [62, p. 14] [*Caturus branchiostegus* Agass. (*Uræus branchiostegus* Collect.) I know only one lower jaw with gill rays. The teeth are close together, the jaw short; the anterior branchiostegal rays wider than the posterior ones. Solenhofen]. Attending to the brief original description, the holotype is the specimen illustrated in LDGSL/614/2/75 (figure 8*a*), watercolour artwork by G. A. H. Köppel in 1837. This very incomplete specimen was part of the collection of Georg Graf von Münster and has not been found.

**Figure 9.**
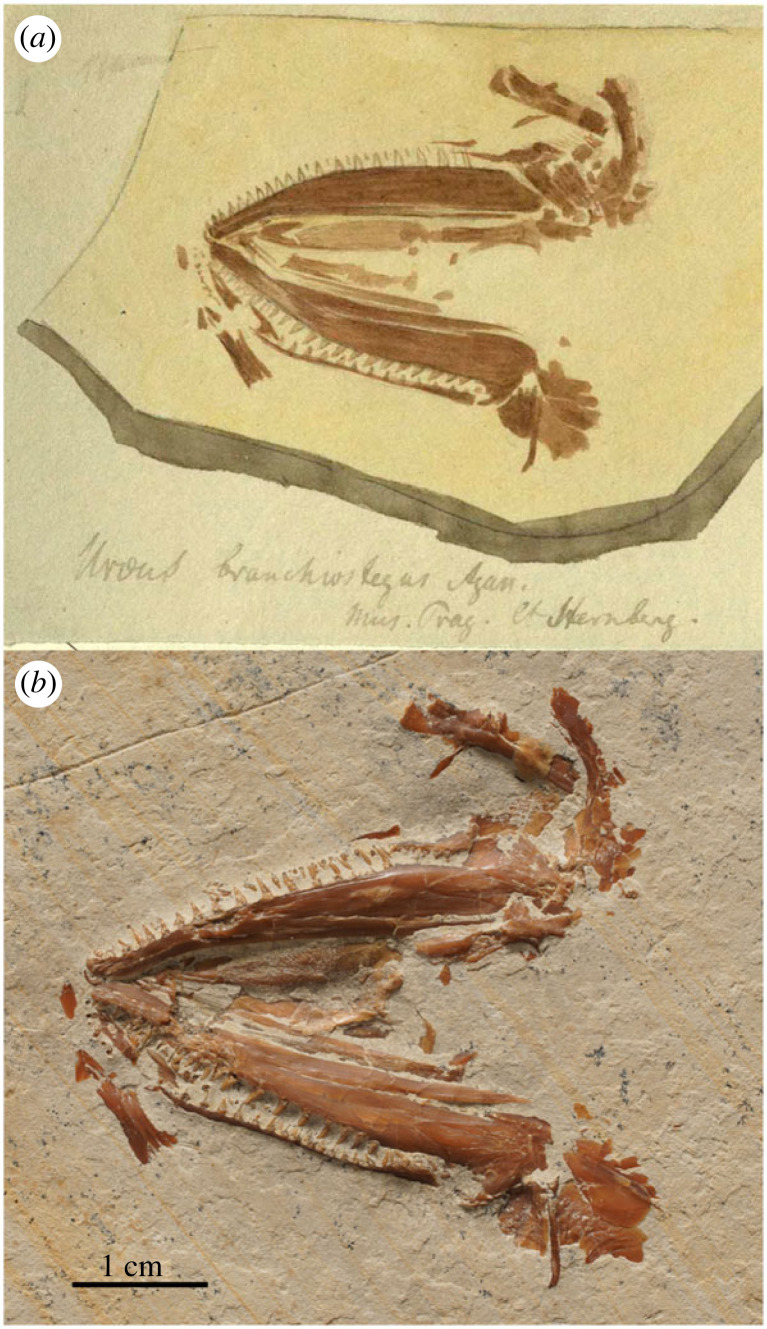
*Caturus* sp. (*a*) LDGSL/614/2/66, illustration of the specimen NMP Uc275 by G. A. H. Köppel in 1837. (*b*) NMP Uc275 from the Upper Jurassic (Tithonian) lithographic limestones of the Solnhofen Archipel (Zandt?), Germany, photograph by B. Ekrt (NMP).

Specimen NMP Uc275 is illustrated in LDGSL/614/2/66, watercolour sketch by Joseph Rössert between 1833 and 1838 (figure 9). Although similarly incomplete as the holotype, branchiostegal rays are not preserved in this specimen, which despite being well-preserved, is very incomplete, including mainly the two lower jaws, left pterygoids, right quadrate and ventral suborbital, and fragments of the preopercles and left hyomandibula. Since Agassiz [62] (see above) based his new species on a single specimen, NMP Uc275 cannot be considered part of the type series.

As already indicated by Woodward [40], it is not possible to distinguish this nominal species which represents a nomen dubium.

### 4.12. *Caturus macrodus* Agassiz, 1834 [62]

The original description is: ‘*Caturus macrodus* Agass. (*Uræus macrodus* Collect.) Espèce encore douteuse, ressemblant beaucoup au *Caturus pachyurus*, mais dont les mâchoires paraissent plus courtes, les dents plus grandes et très -rapprochées les unes des autres. Eichstædt’ [62, p. 14] [*Caturus macrodus* Agass. (*Uræus macrodus* Collect.) A still doubtful species, closely resembling *Caturus pachyurus*, but whose jaws appear shorter, the teeth larger and very close together. Eichstätt]. Two specimens from Solnhofen in the collection of Georg Graf von Münster were illustrated by G. A. H. Köppel in 1837 (LDGSL/614/2/69 and LDGSL/614/2/70). None of the specimens has been located, and it is nonetheless doubtful if they correspond to the type specimen studied by Agassiz, the provenance of which is indicated Eichstätt.

*Caturus macrodus* has been regarded as a junior synonym of *Caturus furcatus* [23,40] or *Amblysemius bellicianus* [28]. In the absence of a type specimen and considering the deficient original description, the taxon is here regarded as a nomen dubium.

### 4.13. *Caturus latus* Münster, 1834 [65]

**Figure 10.**
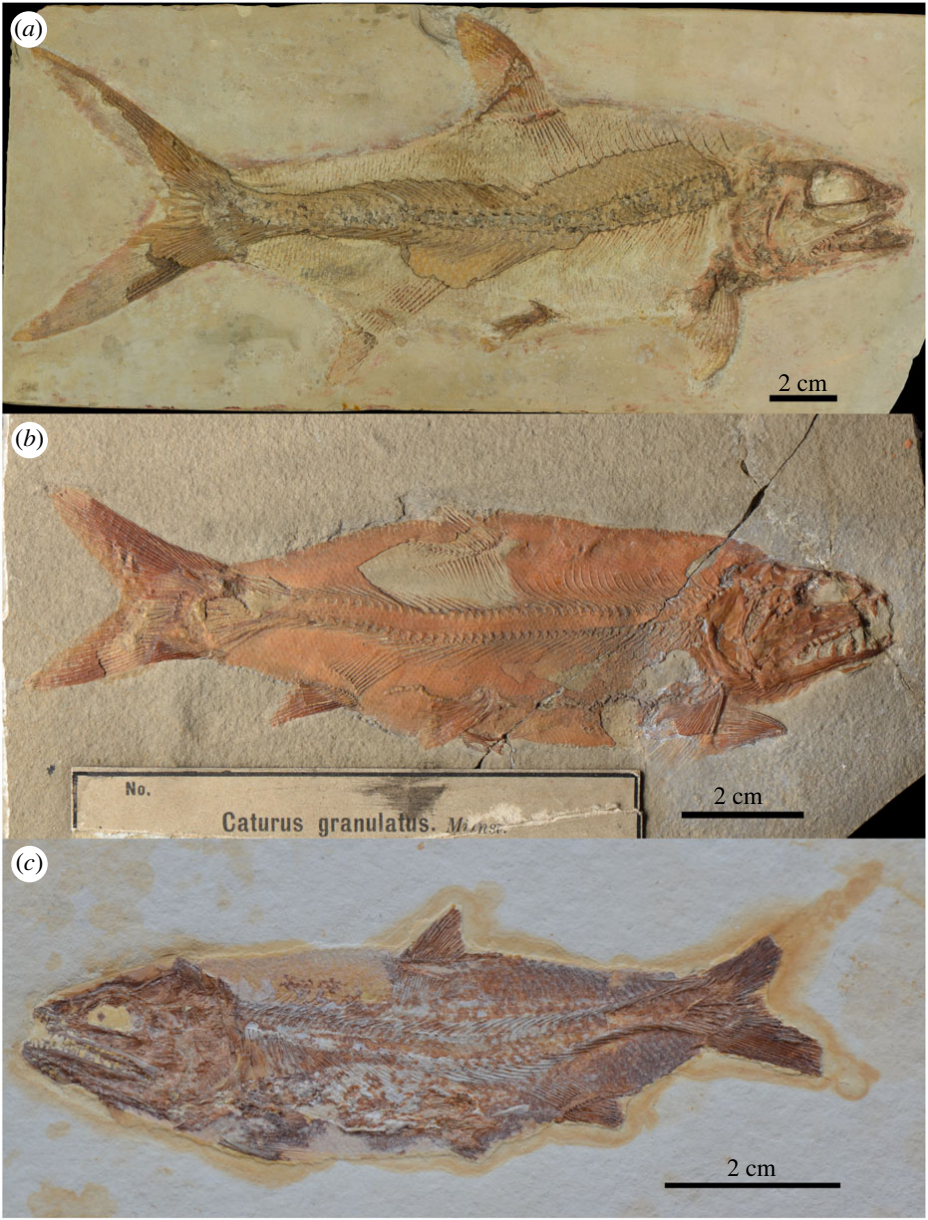
Late Jurassic nominal species previously considered junior synonyms of *Caturus furcatus* or species of *Amblysemius*, which probably represent distinct taxa. (*a*) *Caturus latus* Münster, 1834 [65], holotype SNSB-BSPG AS VIII 263 from the Tithonian of Solnhofen, Germany. (*b*) *Caturus granulatus* Münster, 1842 [61], holotype SNSB-BSPG AS VII 1139 from the Kimmeridgian of Kelheim, Germany. (*c*) *Caturus ferox* Winkler, 1862 [66], holotype TM 6901 from the Tithonian of Solnhofen, Germany. Photographs by R. Winter (LMU).

Holotype SNSB-BSPG AS VIII 263 from Solnhofen (figure 10*a*). The species name was proposed by Münster, 1834, with a very brief and insufficient description. However, acknowledging the authority of Münster, Agassiz (Agassiz 1839) published an illustration of the holotype, which had been designated by Münster in 1834.

*Caturus latus* has been considered as a junior synonym of *Caturus furcatus* [23,28,40]. However, although the holotype is imperfectly preserved, it is a quite complete fish and our recent revision of this and other referred specimens supports the validity of this species, which is currently under study.

### 4.14. *Caturus angustus* Münster, 1839 [67]

Valid as *Anaethalion angustus* according to Münster [61] with neotype JME-SOS2271 from Eichstätt, Germany, designated by Nybelin [68] as ‘Eichstätt III’ [69].

### 4.15. *Caturus angustissimus* Münster, 1839 [67]

Valid as *Anaethalion angustissimus* according to Münster [61] with neotype NHMUK PV OR 37901 from Solnhofen, Germany, designated by Nybelin [68].

### 4.16. *Caturus ovatus* Münster, 1839 [67]

The holotype SNSB-BSPG AS VII 266, from Kelheim (most probably Kapfelberg), Germany, is an incomplete fish preserved in left lateral view, consisting of the almost complete postcranium including all of the fins, except the pectorals. The species is listed as a junior synonym of *C. furcatus* by Lambers [23,28]. However, the specimen was distinguished as a species of the teleost genus *Pholidophorus* by Wagner [54], under the name *Pholidophorus falcifer*.

Although the referral of this species to *Pholidophorus* should be confirmed with further study, our direct observations on the type specimen confirm that it is a teleost and the name is provisionally referred to as ‘*Pholidophorus*’ *ovatus* [67]. In any case, the name *Pholidophorus falcifer* Wagner, 1863 [54], although it remains available (ICZN Art. 10.6), it is invalid because it is an objective synonym of *Caturus ovatus* Münster, 1839 [67] (ICZN Art. 23, glossary).

### 4.17. *Caturus granulatus* Münster, 1842 [61]

The holotype SNSB-BSPG AS VII 1139, from Kelheim (most probably Kapfelberg), Germany, is a complete fish imperfectly preserved in right lateral view (figure 10*b*). The original description is very brief: ‘*C. granulatus*, ein 5 1/2’ langer, vorzüglich gut erhaltener Fisch, mit sehr kleinen granulierten Schuppen, fast wie die Chagrin-Haut eines Hayes; ohne sichtbare Wirbel; der Kopf sehr klein, mit äusserst grossen konischen Zähnen’ [61, p. 44] [*C. granulatus*, a 5 1/2’ long fish, excellently preserved, with very small granulated scales, almost like the chagrin skin of a shark; without visible vertebrae; the head very small, with extremely large conical teeth].

Woodward [40] was doubtful about the taxonomic validity of *Caturus granulatus* and Lambers [23,28] differentiated SNSB-BSPG AS VII 1139 from *Caturus*, and thought it represents the genus *Amblysemius* regarding the species as a junior synonym of his *Amblysemius pachyurus* (see above discussion on *Amblysemius*). Our study of SNSB-BSPG AS VII 1139 supports the validity of *Caturus granulatus* Münster, 1842 as a distinct species of *Amblysemius* which is currently under study.

### 4.18. *Caturus obovatus* Münster, 1842 [61]

Münster did not figure a type specimen or indicate a collection number, and the brief original description of this nominal taxon is not diagnostic: ‘*C. obovatus*, ein 3 1/2’ langer, ebenfalls ganz vollständiger Fisch; er hat eine länglich-eiförmige Gestalt, einen grossen Kopf, kleine konische Zähne, feine glatte Schuppen; Wirbel sind in keiner der beiden Hälften sichtbar’ [61, p. 44] [*C. obovatus*, a fish 3.5 in long, also quite complete; it has an elongated ovoid shape, a large head, small conical teeth, fine smooth scales; vertebrae are not visible in any of the two halves].

Specimen MB.f.12850 (figure 5*c*) is the only specimen found with the label *Caturus obovatus* in the old collection of Georg Graf von Münster. Moreover, the fish matches the few features included in the original description of this taxon. In particular, it has exactly the same length (8.5 cm = 3.5 in). Therefore, MB.f.12850 is here designated holotype of *Caturus obovatus* Münster, 1842 (ICZN Arts. 72.4.1.1 and 74.7).

The species has been considered as a junior synonym of *Caturus furcatus* [23,28,40], but MB.f.12850 is extremely similar to the holotype of *C. macrurus* NMP Uc74 (figure 5*b*), which might represent a distinct taxon. If the validity of this species is confirmed, the name *C. macrurus* Agassiz, 1833 [41] has priority over *C. obovatus* Münster, 1842 [61].

### 4.19. *Caturus intermedius* Münster, 1842 [61]

No type specimen could be found, and the original description is insufficient to distinguish the taxon: ‘*C. intermedius*. Auch bei diesem Fisch sind die Wirbel nicht zu erkennen, obgleich, wie bei den vorhergehenden Arten, die Wirbel-Fortsätze u. s. w. sichtbar sind. Ein Theil des Kopfes fehlt; der Körper ist schmal; die glatten Schuppen sind gross u. s. w.’ [61, p. 44] [*C. intermedius*. In this fish, too, the vertebrae are not visible, although, as in the preceding species, the vertebral processes etc. are visible. A part of the head is missing; the body is narrow; the smooth scales are large, etc.]. Wagner [54] studied the type specimen and referred it to *Pholidophorus radians* Agassiz, 1834 [62].

Woodward [40] placed *Caturus intermedius* under synonymy with *Pholidophorus macrocephalus* and Lambers [23,28] placed it under synonymy with *C. furcatus*. Neither Münster [61] nor Wagner [54] gives any indication about the type specimen, not even its provenance. In the absence of a type specimen and considering the deficient original description, and the confusing appreciation of this taxon by previous authors, who understood it as either a teleost or a caturid, the taxon is here regarded as a nomen dubium.

### 4.20. *Caturus brevicostatus* Münster, 1842 [61]

With only one exception, this nominal species has never been mentioned again after the original description, which is very vague and there is no indication of a type specimen or locality: ‘*C. brevicostatus*, zeichnet sich durch sehr kurze Flossen und Rippen oder Stachel-Fortsätze aus. Auch an dieser Art fehlen die Wirbel. Sie ist dem *Megalurus brevicostatus* ähnlich, von welchem sie aber schon durch die gegabelte Schwanz-Flosse hinreichend abweicht’ [61, p. 44] [*C. brevicostatus*, is characterized by very short fins and ribs or spiny appendages. This species also lacks vertebrae. It is similar to *Megalurus brevicostatus*, from which it differs sufficiently by the forked tail fin].

*Caturus brevicostatus* was unknown to Woodward [40, p. 350] and was cautiously listed as a junior synonym of *C. furcatus* by Lambers [23,28]. Münster [61] gives no indication of the specimen examined and it has not been possible to find any potential holotype. Therefore, the species is regarded as a nomen dubium.

### 4.21. *Caturus angustus* Agassiz, 1844 [49]

The name *Caturus angustus* Agassiz, 1844 is a homonym of *Caturus angustus* Münster, 1839 [67], but both names are valid because they are currently not cogeneric. The latter taxon is the genotype of *Anaethalion* White, 1938 [70] (see §4.14).

The original and only known description of *C. angustus* Agassiz, 1844 is very limited: ‘Espèce très-allongée, remarquable par le développement excessif des fulcres du lobe supérieur de la caudale’ [49, p. 118] [Very elongated species, remarkable for the excessive development of the fulcrum of the upper lobe of the caudal fin]. The fish was later studied by Woodward [71] who provides a complete description of a specimen from the Portlandian, Tithonian, Garsington, Oxford, in the Worcester Museum. Woodward fixed this specimen as the holotype. Although Woodward's description of the specimen is rather detailed, it is not clear if *C. angustus* Agassiz, 1844 is a species of *Caturus* and a detailed study of the type specimen is necessary to evaluate the validity of this taxon.

Two unpublished artworks representing the holotype designated by Woodward and its counterpart have been located. The counterpart was illustrated by Joseph Dinkel between 1834 and 1839 (LDGSL/614/2/65). The specimen was part of the collection of William Buckland, but it has not been located in Oxford University Museum of Natural History and the whereabouts of the specimen are unknown (H. Ketchum 2022, personal communication). The watercolour of the holotype (LDGSL/616/2/57/2) was also done by Joseph Dinkel, but later in 1860. However, the artwork is accompanied by a reference to ‘“Recherches sur les Poissons Fossiles” (1833–1843/1844), Vol 2, pt2, [p118]’, indicating that Agassiz studied both part and counterpart of the fish. The whereabouts of the holotype specimen in the Worcester Museum are also unknown.

*Caturus angustus* Agassiz, 1844 was accepted as valid by Woodward [40], but it is not included in the work of Lambers [23,28]. Pending the localization and study of the type specimen, the taxon is provisionally regarded as a nomen dubium.

### 4.22. *Caturus driani* Thiollière, 1851 [47]

The original description is not sufficient to distinguish the species: ‘C'est l'espèce inédite que j'indiquais sous le n. 4 de ma première liste, sans oser en déterminer le genre. A celle époque je n'avais pas encore eu l'occasion de me convaincre de toute l'étendue dans laquelle varient les formes et même la position de la dorsale chez les *Caturus*. Je ne puis aujourd'hui admettre comme base d'une coupe générique, la circonstance que cette nageoire est placée encore un peu plus en avant des ventrales, qu'elle ne l'est déjà chez le *C. velifer*; lorsque d'ailleurs aucune autre modification un peu apparente n'existe dans le reste du corps. Ce qui achève de différencier cette espèce de la précédente, c'est que la caudale n'est pas plus grande que chez le *C. furcatus*; tandis que la dorsale, les ventrales et les pectorales l'étant bien davantage, il n'est pas possible non plus de supposer que ce ne soit là qu'une variété de celle dernière. Ce que je disais des proportions des vertèbres ne doit s'entendre que de leurs apophyses, car, je le répète, pas plus pour celui-ci que pour les autres *Caturus* trouvés à Cirin, le corps des vertèbres n'existe’ [47, 37–38] [It is the unpublished species which I indicated under n. 4 of my first list, without daring to determine its genus. At that time, I had not yet had the opportunity to convince myself of the full extent to which the forms and even the position of the dorsal fin varies in the *Caturus*. I cannot now admit as the basis of a generic section the circumstance that this fin is placed even a little further forward of the ventrals than it already is in *C. velifer*; when, moreover, no other slightly apparent modification exists in the rest of the body. What completes the differentiation of this species from the preceding one, is that the caudal is not larger than in *C. furcatus*; whereas the dorsal, ventral and pectoral being much larger, it is not possible either to suppose that this is only a variety of the latter. What I said about the proportions of the vertebrae must be understood only in terms of their apophyses, for, I repeat, no more for this one than for the other *Caturus* found at Cerin, the body of the vertebrae exists]. In this description Thiollière merely describes the morphological differences between his new species and *C. velifer*, which is not a species of *Caturus* (see below).

*Caturus driani* has been considered valid [23,28,40]. However, Thiollière did not designate a holotype or figured a specimen of his *C. driani*, and it is therefore not possible to be certain about the validity of this nominal taxon which is a nomen dubium. While acknowledging the uncertainty about the validity of Thiollière's nominal species, Saint-Seine [52] proposed to maintain the name of *C. driani* to designate a species of Cerin, which he distinguished from *C. velifer* by elasmoid scales in the former species as opposed to the rhomboid scales in the latter. Although Saint-Seine [52] did not fix a type specimen of his *C. driani*, his description is mainly based on the specimen MHNL20015190, which is indistinguishable from *C. furcatus*.

### 4.23. *Caturus velifer* Thiollière, 1851 [47]

Holotype MHNL20015388 from Cerin, France. The species is valid, but it is not a species of *Caturus*. It has been excluded from this genus as ‘*Pholidophorus*’ *velifer* by Vetter [72, p. 111], and it is currently under study.

### 4.24. *Amblysemius bellicianus* Thiollière, 1851 [47]

Holotype MHNL20015164 from Cerin, France (figure 4). Our ongoing research on this taxon based on the type and other referred specimens confirms its validity. *Amblysemius bellicianus* Thiollière, 1851 is the type species of *Amblysemius* Agassiz, 1844 (see above).

### 4.25. *Strobilodus giganteus* Wagner, 1851 [38]

Valid taxon (see §3.1).

### 4.26. *Caturus ferox* Winkler, 1862 [66]

The holotype TM 6901 from Solnhofen, Germany, is an almost complete and well-preserved specimen in left lateral view (figure 10*c*). The excellent and detailed description by Winkler [66] is literally reproduced and translated in the electronic supplementary material, File.

*Caturus ferox* has been considered as a junior synonym of *Caturus furcatus* [23,28,40]. However, our recent revision of the holotype supports the validity of this species, which is currently under study.

### 4.27. *Caturus brevis* Winkler, 1862 [66]

Holotype TM 6899 from Solnhofen, Germany [72,73]. Vetter [72] noticed that this specimen is not a *Caturus* and suggested it could be and exemplar of *Pholidophorus micronyx*. The specimen TM 6899, with rhomboid ganoid scales, is certainly not a *Caturus* and needs new study.

### 4.28. *Caturus cyprinoides* Wagner, 1863 [54]

The original description is not sufficient to solve the taxonomic status of this nominal species: ‘Nach 3 sehr schönen Doppelplatten aus der Eichstädter Sammlung habe ich diese Art aufgestellt, die nach ihrer Grösse und breiten Leibesform die grösste Aehnlichkeit mit *C. latus* hat, sich aber sogleich dadurch unterscheidet, dass sie keine ringförmigen Hohlwirbel hat, sondern dass zwischen den kurzen oberen und untern Halbwirbeln ein breiter, glatter, unabgetheilter Zwischenraum liegt, den ehemals die weiche Rückensaite erfüllte. Dazu kommt nun noch, dass die Rückenflosse kürzer und die Schnauze spitziger ist. Das Gebiss ist sehr kräftig. Länge 9″, Rumpfbreite 2″ 71/2″′, Rückenflosse 1″ 2″′ hoch, Schwanzflosse am Aussenrande 2″ 8 bis 11″′. Ein kleineres Exemplar ist nur 7″ lang’ [54, pp. 702–703] [After 3 very beautiful double plates from the Eichstätter collection I have setup this species, which has the greatest resemblance to *C. latus* according to its size and broad body shape, but immediately differs in that it has no ring-shaped hollow vertebrae, but that between the short upper and lower hemivertebrae is a wide, smooth, unhealed space, which formerly filled the soft dorsal string. In addition, the dorsal fin is shorter and the snout more pointed. The dentition is very strong. Length 9″, trunk width 2″ 71/2″′, dorsal fin 1″ 2″′ high, caudal fin at outer margin 2″ 8 to 11″′. A smaller specimen is only 7″ long].

The species has been placed under synonymy with *Caturus furcatus* [23,28,40]. The holotype of *C. cyprinoides* SNSB-BSPG AS V 514 from Eichstätt is a complete specimen rather well preserved in part and counterpart. Our revision of the holotype and comparison with other material indicate it is most probably a junior synonym of *C. latus*.

### 4.29. *Caturus contractus* Wagner, 1863 [54]

Wagner based this species on a single specimen from Solnhofen, Germany, that could not be found in the collections of the BSPG and was probably lost during WWII. Wagner [54] distinguished this species mainly on the body proportions of this specimen compared to three specimens of *C. pachyurus* and the holotype of *C. granulatus*. ‘So ähnlich auch diese Art, von der nur ein einziges Exemplar von Solenhofen vorliegt, dem *C. pachyurus* ist, so unterscheidet sie sich doch von letzterem sehr erheblich durch den gedrängteren und weit robusteren Körperbau, wie diess die vorhin angeführten Maasse ausweisen. Auch ist die hohe Rückenflosse etwas mehr vorgerückt und der Kopf verhältnissmässig grösser und kräftiger. Die Zähne in beiden Kiefern sind zahlreich’ [54, pp. 705–706] [As similar as this species, of which only one specimen from Solenhofen is available, is to *C. pachyurus*, it differs from the latter very considerably by the more compact and far more robust body shape, as the previously mentioned measurements show. Also, the high dorsal fin is somewhat more advanced and the head is relatively larger and stronger. The teeth in both jaws are numerous]. The few measurements provided by Wagner indicate differences between the compared species. *Caturus contractus* would have a comparatively deeper body, larger head and more anteriorly placed dorsal fin than *C. pachyurus*.

**Figure 11.**
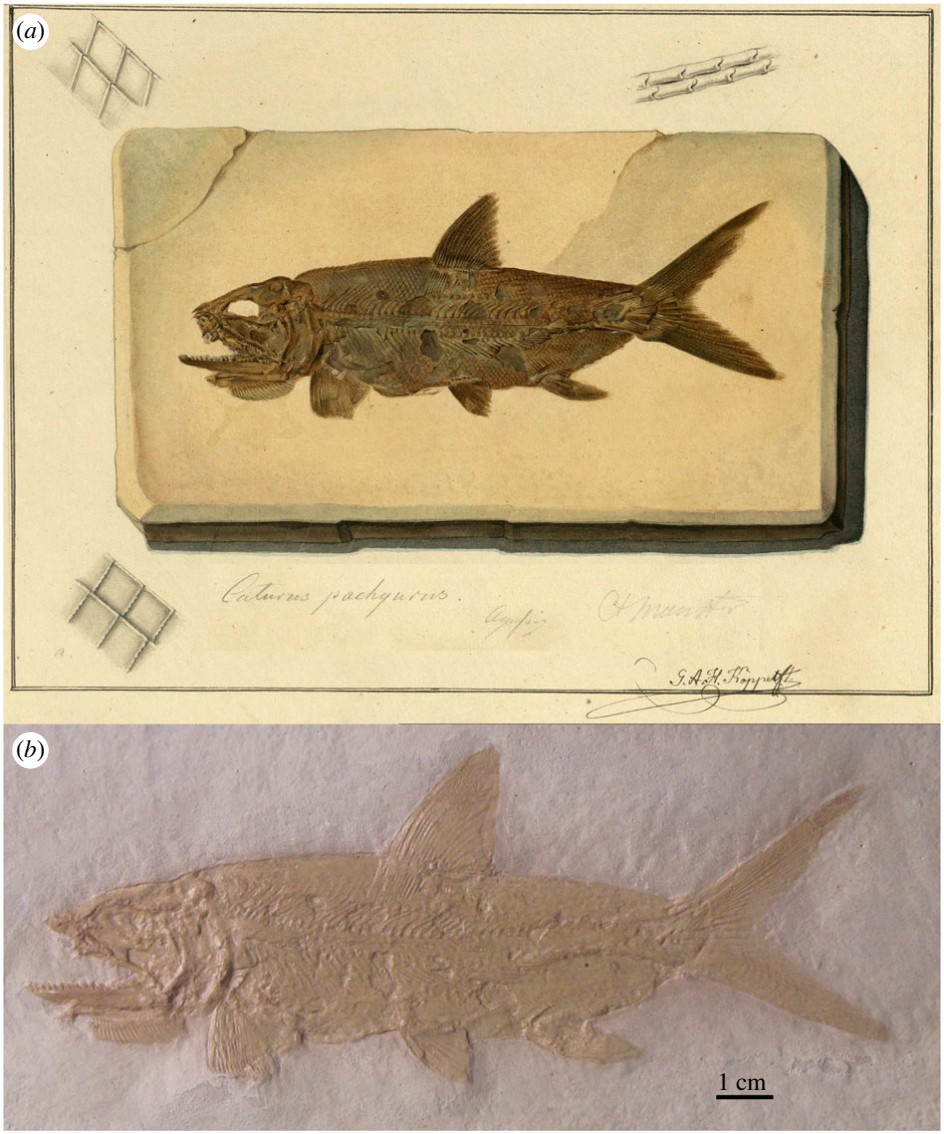
*Caturus contractus* Wagner, 1863 [54]. (*a*) LDGSL/614/2/77, illustration of the type specimen by G. A. H. Köppel in 1837. (*b*) Photograph of NHMUK PV OR 49127, cast of the holotype from the Upper Jurassic (Tithonian) lithographic limestones of Solnhofen, Germany.

The unpublished drawing LDGSL/614/2/77 (figure 11*a*) intended for Agassiz's ‘Recherches sur les Poissons Fossiles' supports the indication by Woodward [40] that NHMUK PV OR 49127 (figure 11*b*) is a cast of the type specimen of *C. contractus* Wagner, 1863. However, the original specimen is lost and NHMUK PV OR 49127 does not show distinct body proportions as proposed by Wagner (1863 #2072). Therefore, *C. contractus* is here considered a nomen dubium.

### 4.30. *Caturus gracilis* Wagner, 1863 [54]

The name was erected by Wagner for a small fish that he considered a variation of *C. macrurus*: ‘Wird von Agassiz folgendermassen characterisirt: “kleine Art von ohngefähr 4″ Länge, untersetzt und mit kräftigem Skelete”. Es liegen von dieser Art viele Exemplare von Solenhofen und Eichstädt vor. Was Agassiz vom Skelete angab, bezieht sich nur auf die Knöchernen Fortsätze der Wirbelsäule, nicht aber auf die Wirbel selbst, indem diese gar nicht zur Ausbildung gelangt sind, sondern zwischen ihren Apophysen nur ein breiter, leerer Raum vorhanden ist. Nach ihrer äussern Form ähnelt der *C. macrurus* zunächst einem kleinen *C. cyprinoides*; die Rückenlinie ist daher ziemlich stark gewölbt, die Bauchlinie weniger. Der Kopf ist zugespitzt und die Kiefer mit zahlreichen Zähnene besetzt. Die grössten Exemplare haben eine Länge von 4″ 6″′ mit einer Breite von 1″ 3–4″′; die kleinsten sind wenig über 3″ lang und von wechselnder Breite, indem der Rumpf bald robuster, bald schmächtiger ist. Letztere Form habe ich in der Sammlung als *C. gracilis* bezeichnet; sie bildet jedoch wegen der Uebergänge in die breitere Form nur eine Varietät’ [54, pp. 706–707] [Is characterized by Agassiz as follows: ‘small species of about 4″ length, stocky and with a strong skeleton’. There are many specimens of this species from Solenhofen and Eichstädt. What Agassiz stated of the skeleton refers only to the bony processes of the vertebral column, but not to the vertebrae themselves, in that these have not reached formation at all, but only a broad, empty space is present between their apophyses. According to its external form the *C. macrurus* resembles at first a small *C. cyprinoides*; the dorsal line is therefore rather strongly arched, the ventral line less. The head is pointed and the jaws are set with numerous teeth. The largest specimens have a length of 4″ 6″′ with a width of 1″ 3–4″′; the smallest are little over 3″ long and of varying width, in that the trunk is sometimes more robust, sometimes more slender. I have called the latter form *C. gracilis* in the collection; however, it forms only one variety because of the transitions into the broader form]. However, the name *C. gracilis* Wagner, 1863 has been considered available and the species is currently under synonymy with *Caturus furcatus* [23,28,40].

Specimen SNSB-BSPG AS I 1247 from Eichstätt is indicated as the holotype of *C. gracilis*. The small complete fish is very poorly preserved in left lateral view, and it has not been possible to confirm its potential synonymy with *C. furcatus* [23,28,40] or the validity as a distinct taxon. The name *C. gracilis* Wagner, 1863 is a homonym of *Caturus gracilis* (Agassiz, 1832) [43]. However, since both nominal taxa are considered nomina dubia, it is not possible to establish whether they are cogeneric and therefore it is useless to propose a new name for the junior homonym.

### 4.31. *Caturus fusiformis* Wagner, 1863 [54]

Wagner [54] indicated this binomen referring to his previous labelling of two specimens in the collection, but without the intention of naming a new taxon. On the contrary, he undoubtedly referred the two so-labelled specimens to *C. elongatus* Agassiz, 1834: ‘Noch näher an das Original-Exemplar schliessen sich 2 kleinere von Solenhofen und Eichstädt, die ich in hiesiger Sammlung als *C. fusiformis* bezeichnete. Bei ihnen verläuft der Rumpf wirklich fast in einer Flucht, indem er von vorn an nach hinten sich ganz allmählig verschmälert; der Kopf ist von gleicher Form wie bei den anderen Exemplaren. Länge 5′, grösste Rumpfbreite 1′ 1″. Man möchte fast eine eigene Art daraus errichten’ [54, p. 704] [Even closer to the original specimen are 2 smaller ones from Solenhofen and Eichstädt, which I labelled in my collection as *C. fusiformis*. In them the trunk is really almost in alignment, narrowing gradually from the front to the back; the head is of the same shape as in the other specimens. Length 5′, greatest trunk width 1′ 1″. One would almost like to create a separate species thereof].

The name *Caturus fusiformis* Wagner, 1863 was not proposed as a new name. It must be treated as a name published as synonym, and it is therefore not available (ICZM Arts. 11.5.2 and 11.6).

### 4.32. *Thlattodus suchoides* Owen, 1866 [74]

The holotype NHMUK PV OR 41386 is a three dimensionally preserved partial skull from the Kimmeridgian of Norfolk, UK. The general shape of the maxilla and the strong dentition made of large marginal teeth with stout conical caps indicate close resemblance with the specimens of *Strobilodus giganteus*, supporting Zittel's [75] referral of Owen's species to the latter genus as *Strobilodus suchoides*.

### 4.33. *Ditaxiodus impar* Owen, 1866 [76]

The holotype NHMUK PV OR 46318, from the Kimmeridgian of Oxfordshire, UK, consists of incompletely preserved maxilla and denary bones with teeth. The remains are very similar to the specimens of *Thlattodus suchoides* and *Strobilodus giganteus*. Owen distinguished the two species from the Kimmeridge Clay *Thlattodus suchoides* and *Ditaxiodus impar* based on the characteristics of their teeth. A thorough analysis of these features is needed to evaluate the validity of these taxa. Pending such a study, the species is here tentatively referred to as *Strobilodus impar*.

### 4.34. *Caturus segusianus* Thiollière, 1873 [53]

The specific name is known from a figure [53, fig. 1, pl. XII], but the species was never described or properly published. Although the name was placed under synonymy with *Caturus driaini* by Woodward [40] and Saint-Seine [52], *C. segusianus* is not an available name (ICZN Arts. 11–12).

The specimen figured under the name *C. segusianus* [53, fig. 1, pl. XII] is MHNL20015190, which has been considered to represent *Caturus driani* by Gervais (in [53]), or *Caturus velifer* by Saint-Seine [52]. The recent study of MHNL20015190 confirms that it is a specimen of ‘*C*.’ *velifer*, which is not a species of *Caturus*.

### 4.35. *Caturus cliftoni* Woodward, 1895 [40]

Holotype NHMUK PV P 6035 imperfect right maxilla, from the Portlandian (Tithonian), Isle of Portland. The holotype is very fragmentary, but the dentition differs from the teeth of *Caturus* because they lack the labiolingually compressed and sharply carinate acrodin caps typical of the latter genus. Other specimens referred to this species by Woodward [40] NHMUK PV P 6034 (right dentary), NHMUK PV P 6034a (middle portion of a maxilla) and NHMUK PV OR 42381 (portion of a left maxilla) from the type locality, and NHMUK PV OR 40719 (right maxilla) from the Kimmeridge Clay in Dorsetshire, show similar dentition to the holotype. The characteristics of the teeth suggest that *C. cliftoni* is a species of *Strobilodus* and is here tentatively referred to the latter genus. *Strobilodus cliftoni* n. comb. should be compared in detail not only with *Strobilodus giganteus* but also with the Kimmeridgean taxa *Strobilodus* (*Thlattodus*) *suchoides* and *Strobilodus* (*Ditaxiodus*) *impar*.

### 4.36. *Caturus deani* Gregory, 1923 [77]

Holotype AMNH FF 6371 (7930) from the Upper Jurassic Jagua Formation (Oxfordian) of Cuba (Iturralde-Vinent and Ceballos Izquierdo, 2015). The species is based on an imperfectly preserved skull. Additional, still unstudied specimens are hosted in the collections of the AMNH and MCZ.

### 4.37. *Catutoichthys olsacheri* Gouiric-Cavalli, 2016 [78]

Holotype MOZ-Pv 3645 from the Tithonian of Los Catutos, Vaca Muerta Formation, Neuquén, Argentina. The species is based on a rather well-preserved postcranium showing distinct features of the Caturoidea.

## 5. Diversity of caturids through time

Although we did not revise the taxonomic status of any caturid species except for those from Upper Jurassic sediments, we collected the available information from the literature to complete an overview of the recognized diversity of these fishes (table 1).

The oldest confident record of a caturid is a disarticulated skull MPCA 632, from the lower Upper Triassic of Río Negro province, Argentina [79]. Typical caturid teeth are described for this specimen, but the shape of the maxilla is very different from that of the maxilla of *Caturus* or *Amblysemius* (A.L.-A., personal observation). Therefore, the fish probably represents a distinct, still undescribed caturid taxon. The provenance of MPCA 632 is indicated as the Upper Triassic Vera Formation of the Los Menucos Group [79]. Recent geochronological studies reposition this sequence in the Upper Permian–Lower Triassic [80]. Based on our current knowledge of the fossil record of the group (table 1), the presence of a caturid in freshwater deposits of that age is extremely surprising. After careful revision of the information about the finding of MPCA 632, its provenance from Los Menucos is doubtful (S. Bogan and I. Cerda 2022, personal communication).

The only named Triassic taxon currently classified in Caturidae is *Eugnathus insignis* Kner, 1866 [81] from the Norian of Seefeld, Austria, which was referred to *Caturus* by Woodward [40]. We have been able to locate the type specimen in the Sammlungs- und Forschungszentrum der Tiroler Landesmuseen, Hall in Tirol, Austria. Although the fish shares an overall similarity with *Caturus*, a detailed study of the type and referred specimens is necessary to confirm its systematic position.

Five Early Jurassic species of Caturidae are currently recognized. Four species from Lyme Regis, UK, were referred to *Caturus* by Woodward [40]: *Pachycormus heterurus* Agassiz, 1839 [20], *Eugnathus chirotes* Agassiz, 1842 [82], *Pachycormus latipennis* Agassiz, 1844 [83] and *Endactis agassizi* Egerton, 1858 [84]. *Conodus ferox* Agassiz, 1844 [58] is a junior synonym of *Caturus chirotes* according to Woodward [40]. The other valid Early Jurassic species is *Caturus smithwoodwardi* White, 1927 [85], which is known from the Lower Jurassic (Toarcian) of Holzmaden, Germany.

Unable to find the type specimen of the poorly defined *Caturus bucklandi* Agassiz, 1844 [49], also known from Lyme Regis, Woodward [40] cautiously indicated that it could be a junior synonym of *C. heterurus*. Similarly, Woodward [40] considered *Caturus meyeri* Agassiz, 1844 [49], from the Lower Jurassic (Pliensbachian) of Werther, North Rhine-Westphalia, Germany, too poorly defined and probably a junior synonym of *Pachycormus curtus*.

Other Early Jurassic fossils originally named in *Caturus* are *C. stenospondylus* Sauvage, 1875 [86], *C. cotteaui* Sauvage, 1875 [86], *C. stenoura* Sauvage, 1875 [86], *C. chaperi* Sauvage, 1883 [87] and *C. retrodorsalis* Sauvage, 1891 [88], all of them from the Pliensbachian/Toarcian of Sainte-Colombe, Yonne, France. Except for the last of these nominal species, all other are pachycormid fishes. *C. stenospondylus*, *C. stenoura* and *C. chaperi* are currently considered synonyms of *Pachycormus macropterus*; *C. cotteaui* is a junior synonym of *Euthynotus intermedius* [40,89–91]. The last species, *C. retrodorsalis*, was transferred to *Pholidophorus* [40,91].

Three species are recognized in the Middle Jurassic: *Caturus pleiodus* Agassiz, 1844 [49], from the mid-Bathonian of Stonesfield, UK, *C. dartoni* Eastman, 1899 [92], from the Bathonian (lower Sundance Formation) of Hot Springs, South Dakota, USA and *C. porteri* Rayner, 1948 [93], from the Callovian of the Oxford Clay at Christian Malford, UK. Almost nothing is known about *C. pleiodus*. Agassiz [49] created this name for some isolated upper jaw, which he distinguished by the number and the shape of its teeth. However, he did not report the number of teeth in the jaw, and did not indicate any specimen. Woodward [94] studied three lower jaw fragments (NHMUK PV P 902 and NHMUK PV P 3730) labelled ‘*Caturus pleiodus*’ by Agassiz, but was not able to identify the genus. Thus, *Caturus pleiodus* Agassiz, 1844 [49] should be treated as a nomen dubium.

An incomplete fish from the currently considered lacustrine Upper Jurassic Lime Fine beds of Songa [95] was identified as *Caturus* sp. by Saint-Seine & Casier [96]. However, the specimen has rhomboid ganoid scales and none of the distinctive features of the Caturoidea [13] is preserved. Therefore, this fossil is not a caturoid. Additionally, several incomplete and fragmentary remains, mainly isolated teeth, ranging from the Bathonian (Middle Jurassic) to the Kimmeridgian (Upper Jurassic) have been identified as *Caturus* sp. [97–104].

Finally, the youngest named species of Caturidae are known from the Lower Cretaceous. *Caturus purbeckensis* originally described as *Strobilodus purbeckensis* Woodward, 1890 [105] and *C. tenuidens* Woodward, 1895 [40], from the Berriasian (Middle Purbeck Beds) of Swanage, UK. A third nominal taxon from the same area, ‘*Caturus*’ *latidens* Woodward, 1918 [106], with holotype NHMUK PV P 6360, was named in *Caturus* with caution. Woodward diagnosed the species based on the morphology of the marginal teeth, which are ‘broad, laterally compressed near the apex, and bluntly pointed’ [106, p. 83]. Therefore, they clearly differ from the characteristic conical teeth with laterally compressed and carinate acrodin caps of caturids. Also based on the shape of the marginal teeth, Woodward suggested that ‘*C*.’ *latidens* could be a species of *Callopterus*, but the shape of the maxilla in NHMUK PV P 6360 rather resembles that of the maxilla of *Strobilodus giganteus* than that of *Callopterus agassizi* (A.L.-A., personal observation). A revision of this species is beyond the scope of this contribution, but based on Woodward [40] we already remove the taxon from the family Caturidae. Somewhat younger, *Caturus tarraconensis* Sauvage, 1903 [107], from the Berriasian-lower Valanginian of El Montsec, Lérida, Spain, is represented with very well preserved, though still poorly studied specimens [108].

## 6. Conclusion

Our revision of the 37 Late Jurassic nominal species previously classified in Caturidae led us to the following conclusions:

— Four nominal species represent unavailable names: *Uraeus pachyurus* Agassiz, 1832; *Amblysemius gracilis* Agassiz, 1844; *Caturus segusianus* Thiollière, 1873; and *Caturus fusiformis* Wagner, 1863. These names should be excluded from synonymy lists.
— Whether due to insufficient information in the original descriptions, lack of diagnostic features in the type material, or the complete lack of type material, 13 nominal species of caturids are here regarded as nomina dubia (table 2).
— Ten species originally named in *Caturus*, or *Uraeus* before this generic name was replaced, have been referred to other actinopterygian genera outside Amiiformes (table 3).
— Besides the type species of *Caturus*, *C. furcatus*, *Amblysemius*, *A. bellicianus* and *Strobilodus*, *S. giganteus*, we have located and examined the type material of four nominal species representing probably distinct valid taxa (table 4). The thorough revision of these species is ongoing research being carried out by the authors.
— Among the Early–Middle Jurassic species, *Uraeus gracilis* Agassiz, 1832 is a nomen dubium and the validity of *Caturus bucklandi* Agassiz, 1843, *Caturus meyeri* Agassiz, 1843 and *Caturus pleiodus* Agassiz, 1844 is also doubtful [40,105].

**Table 2.** List of Late Jurassic caturoid nominal species here regarded as nomina dubia.

nominal species	type specimen	type locality
*Uraeus nuchalis* Agassiz, 1833	unknown	Solnhofen, Germany
*Uraeus pachyurus* Agassiz, 1833	holotype MCZ VPF-6262	Solnhofen, Germany
*Caturus elongatus* Agassiz, 1834	lost	Solnhofen, Germany
*Caturus maximus* Agassiz, 1834	lectotype CAM SM 11115	Solnhofen, Germany
*Caturus microchirus* Agassiz, 1834	holotype CAM SM 11118	Solnhofen, Germany
*Caturus branchiostegus* Agassiz, 1834	lost	Solnhofen, Germany
*Caturus macrodus* Agassiz, 1834	lost	Eichstätt, Germany
*Caturus intermedius* Münster, 1842	unknown	unknown
*Caturus brevicostatus* Münster, 1842	unknown	unknown
*Caturus angustus* Agassiz, 1844	unknown	unknown
*Caturus driani* Thiollière, 1851	unknown	Cerin, France
*Caturus contractus* Wagner, 1863	lost	Solnhofen, Germany
*Caturus gracilis* Wagner, 1864	SNSB-BSPG AS I 1248	Eichstätt, Germany

**Table 3.** List of nominal species originally named in *Caturus* (*Uraeus*), which are currently classified in other taxa.

nominal species	current taxonomic status	reference
*Uraeus macrocephalus* Agassiz, 1833	*Siemensichthys macrocephalus*	Arratia [51]
*Uraeus microlepidotus* Agassiz, 1833	‘*Furo*’ *microlepidotes*	Lambers [57]
*Caturus angustus* Münster, 1839	*Anaethalion angustus*	Münster [61]
*Caturus angustissimus* Münster, 1839	*Anaethalion angustissimus*	Münster [61]
*Caturus ovatus* Münster, 1839	‘*Pholidophorus*’ *ovatus*	Wagner [54]
*Caturus velifer* Thiollière, 1851	‘*Pholidophorus*’ *velifer*	Vetter [72]
*Caturus brevis* Winkler, 1862	‘*Pholidophorus*’ sp.	Lütken [73]
*Caturus cliftoni* Woodward, 1895	*Strobilodus cliftoni*	this work
*Thlattodus suchoides* Owen, 1866	*Strobilodus suchoides*	Zittel [75]

**Table 4.** List of Late Jurassic nominal species which are currently under study and probably represent distinct taxa (figures 5 and 10). The species previously considered junior synonyms of *Caturus furcatus* are indicated with an asterisk (*).

nominal species	type specimen	type locality
*Caturus macrurus (Agassiz, 1833)	NMP Uc74	Kelheim, Germany
*Caturus latus Münster, 1834	SNSB-BSPG AS VIII 263	Solnhofen, Germany
*Amblysemius granulatus* Münster, 1842	SNSB-BSPG AS VII 1139	Kelheim, Germany
*Caturus ferox Winkler, 1862	TM 6901	Solnhofen, Germany

**Figure 12.**
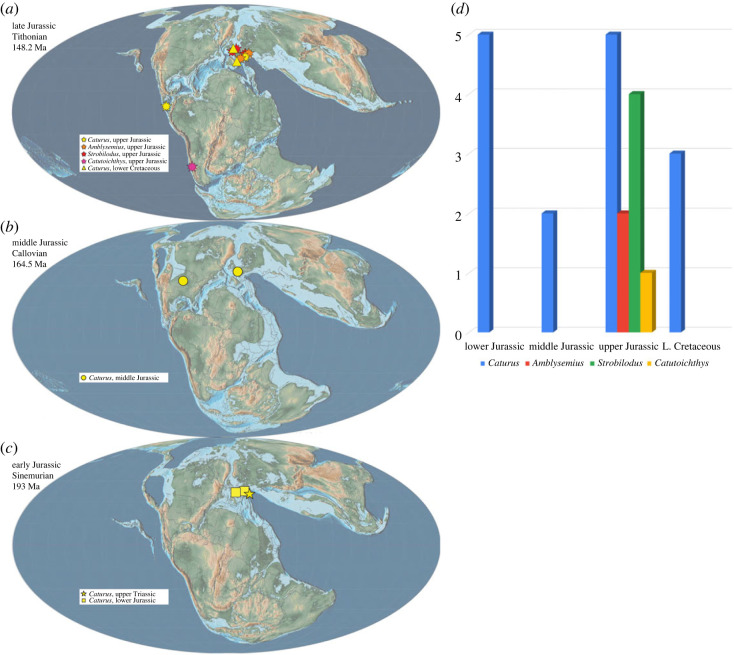
Graphic representation of the known fossil record of caturoids, except the species of *Liodesmus*. (*a*–*c*) Approximate palaeogeographic distribution of caturoid fossil records; palaeomaps modified from [107,109]. (*d*) Diversity of caturoids through time; vertical axis: number of species.

The scarcity of taxonomic studies on caturoids in recent decades does not allow conclusions to be drawn with certainty. However, according to the information gathered regarding the fossil record of Caturoidea, the group is almost restricted to the Jurassic and earliest Cretaceous, with a possible oldest record in the Norian (Late Triassic). The simple graphic representation of the information summarized in table 1 suggests a significant diversification during the Late Jurassic (figure 12). This diversification is not only indicated by the increase in the number of taxa, but also by the dispersal of the group outside Europe, which had already begun in the Middle Jurassic.

The potential diversification of caturoids during the Late Jurassic needs further study and we hope this contribution will stimulate research in that direction. The Early Jurassic species of *Caturus* need thorough revision and, besides the already named species listed in table 4, at least two new Late Jurassic taxa have been identified during this study.

## Data Availability

Additional data supporting this article have been uploaded as part of the electronic supplementary material [110].
